# Cocrystal Engineering of Conjugated Polymer Blends via External Electric Field for Enhanced Charge Transport

**DOI:** 10.1002/advs.202520457

**Published:** 2026-01-07

**Authors:** Yanan Guo, Hao Zheng, Juan Peng

**Affiliations:** ^1^ State Key Laboratory of Molecular Engineering of Polymers Department of Macromolecular Science Fudan University Shanghai China

**Keywords:** charge transport properties, cocrystallization, conjugated polymer blends, external electric field, solution‐state aggregation

## Abstract

Cocrystal engineering that combines different components into cocrystals renders the newly formed materials with innovative and multifunctional properties. However, this strategy is rather limitedly explored in conjugated polymer blends. Herein, we report, *for the first time*, the investigation into the external electric field (EEF)‐induced cocrystallization (i.e., cocrystals of two components) in conjugated polymer blends: poly(3‐butylthiophene) (P3BT) and poly[3,3'''‐dialkyl‐quaterthiophene]s (PQTs) with various alkyl side‐chain lengths (PQT‐C6, PQT‐C8, and PQT‐C10) for significantly improved charge mobilities, and correlate their different cocrystalline and phase‐separated structures (i.e., producing two different crystals from two components) strongly to the aggregation in the solution. Specifically, all three P3BT/PQT blends display bimodal distributed aggregates in the solution and form phase‐separated structures in the as‐cast film. Upon EEF strategy, P3BT/PQT‐C6 and P3BT/PQT‐C8 co‐aggregate in the solution and self‐assemble into cocrystals in the film at the increased EEF strength, demonstrating the cocrystal‐facilitated charge transport in organic field‐effect transistors (OFETs). Conversely, P3BT/PQT‐C10 retain bimodal distribution of aggregates in the solution and thus phase‐separated structures in the film throughout the EEF process. As such, this work demonstrates the robustness of EEF to craft cocrystals in conjugated polymers for the enhancement of charge mobilities, which may facilitate their application in a wide range of optoelectronic devices.

## Graphical abstract

We report, for the first time, an external electric field (EEF) strategy to achieve co‐aggregation of conjugated polymer blends (i.e., poly(3‐butylthiophene) and poly[3,3'''‐dialkyl‐quaterthiophene]s) in the solution and thus their cocrystals in thin films, demonstrating cocrystal‐improved charge mobilities due to the synergy of two components.

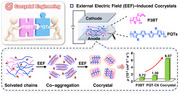

## 1 Introduction

Cocrystal engineering, combining two or more components through non‐covalent interactions (e.g., *π–π* interaction, charge‐transfer interaction, hydrogen bonding), has proven to be an essential and effective strategy in materials and chemistry science [1, 2, 3, 4]. Compared with single‐component systems with their own intrinsic properties, this strategy can render cocrystals with innovative and multifunctional properties via intermolecular collaborative effects [5]. For example, organic small molecules, dibenzotetrathiafulvalene (donor) and tetracyanobenzene (acceptor), can readily aggregate into cocrystals, which exhibit a 2D face‐to‐face stacking and demonstrate a remarkable photothermal conversion efficiency of 18.8 % under near‐infrared irradiation [6]. Moreover, poly(_L_‐lactic acid) (PLLA) and poly(_D_‐lactic acid) (PDLA) with different chirality characteristics are able to cocrystallize into stereocomplex, displaying enhanced mechanical strength and thermomechanical properties [7]. Clearly, benefitting from the rapid development of crystal engineering and material science, organic cocrystals provide an efficient way to craft multifunctional materials with tailored characteristics.

Notably, compared with great advances in cocrystals of small molecules and traditional flexible polymers, the investigation into cocrystallization in semi‐rigid conjugated polymers is rather limited [8, 9, 10, 11]. Conjugated polymers possess distinct rigid backbones and flexible alkyl side chains, coupled with anisotropic intermolecular interactions [12, 13]. These characteristics result in significant challenges in controlling the crystallization kinetics (e.g., nucleation and growth processes) and structures of conjugated polymers, particularly for their blended systems [14, 15, 16]. To achieve cocrystals in such systems requires structural similarity and comparable potential energy [17]. Beyond these requirements, the cocrystallization process in conjugated polymer blends is also highly sensitive to their different crystallization kinetics and crystallization induction between them. Therefore, it remains challenging to form conjugated polymer cocrystals and only limited pairs have been reported [8, 9, 10].

It is known that solution‐state aggregation of conjugated polymers serves as a crucial bridge linking their molecular structures and solid‐state packings [18, 19, 20, 21, 22]. Therefore, it is vital to understand how the aggregation of conjugated polymer blends in the solution affects the formation of cocrystallization in the film. However, this has been rarely explored. Recently, the external electrical field (EEF) strategy has garnered significant attention as a non‐contact approach to control the solution aggregates of conjugated polymers [23, 24, 25, 26]. Because conjugated polymers possess local dipole moments and polarizability, the EEF can assist in overcoming the energy barrier of chain conformation changes, leading to diverse aggregates in the solution [26, 27]. Surprisingly, despite robust characteristics of the EEF strategy, its use for producing conjugated polymer cocrystals and improving their charge transport properties has yet to be explored. Clearly, the ability to craft more conjugated polymer cocrystals to unravel the relationships between their solution‐state aggregation and solid‐state packing can provide deep fundamental insights for their use in high‐performance optoelectronics.

Herein, we report, *for the first time*, an EEF strategy to achieve the cocrystals in a series of conjugated polymer blends for the enhancement of charge transport properties. Specifically, poly(3‐butylthiophene) (P3BT) and poly[3,3'''‐dialkyl‐quaterthiophene]s (PQTs) with various alkyl side‐chain lengths (i.e., hexyl, octyl, and decyl, denoted as PQT‐C6, PQT‐C8, and PQT‐C10, respectively) were chosen as model systems. These three P3BT/PQT‐C6, P3BT/PQT‐C8, and P3BT/PQT‐C10 blends displayed phase‐separated structures (i.e., forming respective P3BT and PQT crystal domains) in their as‐cast films without EEF. After exposing these three blends to EEF treatment, both P3BT/PQT‐C6 and P3BT/PQT‐C8 blends formed cocrystals at an increased EEF strength of 8 and 15 kV/cm, respectively. Importantly, these cocrystal films exhibited much higher charge mobilities than their phase‐separated structures and the corresponding homopolymers, underpinning the concept of cocrystal‐improved charge transport. In contrast, the P3BT/PQT‐C10 blend remained phase separated throughout the EEF process and had only slightly increased charge mobilities compared to the initial as‐cast film due to the increased film crystallinity. These cocrystalline and phase‐separated structures in P3BT/PQT thin films depended sensitively on their aggregation behaviors in the solutions. As such, this work offers a robust route to achieve the cocrystallization in conjugated polymer blends and assess the strong connection among their solution‐state aggregation, cocrystals in the thin film, and carrier transport characteristics to strengthen our fundamental understanding of conjugated polymers.

## 2 Results and Discussion

### 2.1 Cocrystalline and Phase‐Separated Structures in P3BT/PQT Blended Films Rendered by External Electric Field (EEF)

**FIGURE 1:**
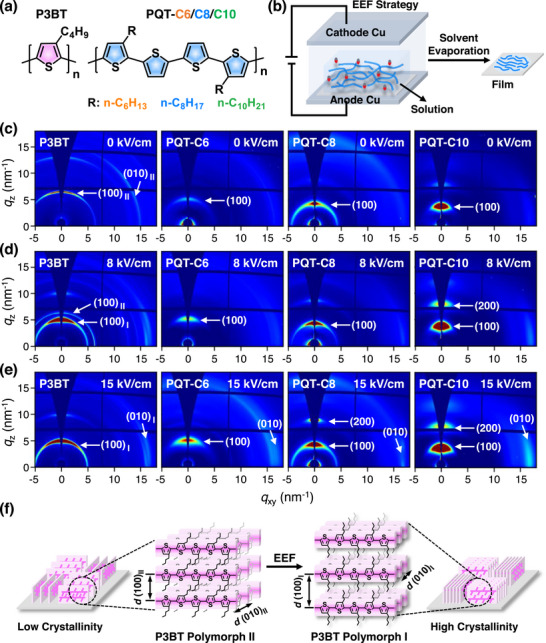
(a) Chemical structures of P3BT and PQTs with different alkyl side‐chain lengths. (b) Schematic of the external electric field (EEF) strategy implemented on the conjugated polymer solution confined between two parallel copper electrode plates, yielding the conjugated polymer film after solvent evaporation. (c‐e) 2D‐GIWAXS images of four homopolymers (P3BT, PQT‐C6, PQT‐C8, and PQT‐C10) produced from 1,2,4‐trichlorobenzene (TCB) under different EEF strengths: (c) 0 kV/cm, (d) 8 kV/cm, and (e) 15 kV/cm. (f) The schematic shows the polymorph transition of P3BT induced by EEF. The initial P3BT film without EEF shows polymorph II in an edge‐on orientation and low crystallinity (left panel). After EEF, it transforms into polymorph I in an edge‐on orientation and high crystallinity (right panel).

P3BT and PQTs are both polythiophene derivatives; they may form cocrystals due to their similar chemical structure. Moreover, alkyl side chains have a great impact on the solution aggregates and thus solid‐state crystalline structures. Therefore, P3BT and PQTs with different side‐chain lengths were chosen as model systems. Specifically, P3BT (*M*_n_ = 3.7 kg/mol) was prepared by Kumada catalyst‐transfer polymerization [28, 29]. PQTs with different alkyl side chains, including PQT‐C6 (*M*_n_ = 15.8 kg/mol), PQT‐C8 (*M*_n_ = 18.9 kg/mol), and PQT‐C10 (*M*_n_ = 20.6 kg/mol), were synthesized by Stille copolymerization [30, 31]. Their molecular structures are depicted in Figure 1a, and their detailed synthesis and characterization are given in Supporting Information (Figures S1–S3 and Table S1). The molecular structures of P3BT and three PQTs were calculated by density functional theory (DFT) (Figure S4). The dihedral angle of thiophene‐thiophene units (*θ*_1_) in P3BT was calculated to be 31.4°. The dihedral angles of the thiophene‐thiophene unit with alkyl side chains (*θ*_1_) and the thiophene‐thiophene unit without alkyl side chains (*θ*_2_) in three PQTs were 13.6°–16.0° and 12.1°–16.7°, respectively. It indicated three PQTs had similar conformation and were more planar than P3BT. Figure 1b describes the schematic of the EEF strategy, in which the P3BT/PQT blended solution was placed between two copper electrode plates with a fixed gap of 5 mm. Different voltages (0–7.5 kV) were applied to the two electrodes, producing the EEF strength (*E*) of 0–15 kV/cm. The solvent gradually evaporated during the EEF treatment. The formed P3BT/PQT blended films were characterized by synchrotron 2D grazing incidence wide‐angle X‐ray scattering (2D‐GIWAXS).

The chain packing and crystalline structures of P3BT and PQT films formed under various *E* were first investigated by 2D‐GIWAXS, in which the scattering of X‐ray was collected in the out‐of‐plane (*q*_z_) and in‐plane (*q*_x,y_) directions (Figure S5). The P3BT film formed without EEF (*E* = 0 kV/cm) exhibited a (100) diffraction ring at *q*_z_ = 6.5 nm^−1^, corresponding to a butyl side‐chain spacing (*d*_100_) of 0.97 nm (Figure 1c). It was ascribed to the P3BT in polymorph II in the edge‐on orientation [32, 33], that is, the P3BT backbone and *π–π* stacking directions were parallel to the substrate, whereas the butyl side‐chain direction was normal to the substrate (Figure 1f, left panel). At *E* = 8 kV/cm, another more intense (100) diffraction peak (*q*_z_ = 5.2 nm^−1^, *d*_100_ = 1.21 nm) appeared, attributed to the polymorph I of P3BT [34, 35, 36]. In this case, two kinds of polymorphs coexisted, in which the intensity of polymorph I was stronger than that of II (Figure 1d). Due to the interdigitated side chains of polymorph II, it had a smaller *d*_100_ than that of polymorph I. Further increasing the *E* to 15 kV/cm yielded pure polymorph I with the disappearance of polymorph II (Figure 1e,f, right panel). By DSC measurement, the endothermic peak of P3BT films formed at *E* = 0 and 15 kV/cm was 121°C and 268°C, respectively, corresponding to the melting temperature of P3BT in polymorph II and I, respectively (Figure S6). These results were aligned well with the 2D‐GIWAXS results and further proved the P3BT polymorph changes rendered by EEF.

As for the other three PQTs, all of them showed (100) diffraction peaks at decreased *q*_z_ (5.0, 4.2, and 3.7 nm^−1^) at *E* = 0 kV/cm, corresponding to an increased *d*_100_ (1.24, 1.48, and 1.69 nm) for PQT‐C6, PQT‐C8, and PQT‐C10, respectively (Figure 1c–e; Table S2). At *E* of 8 and 15 kV/cm, their (100) peaks became stronger with their *d*_100_ nearly unchanged, indicative of increased film crystallinity. These three PQTs showed the melting of side chains (76°C–87°C) and main chains (141°C–157°C) at *E* = 0 kV/cm (Figure S6). At E = 15 kV/cm, the side‐chain melting of three PQTs disappeared, leaving behind their main‐chain melting (141°C–155°C). A possible reason is that the enhanced crystallization of the three PQTs backbones suppressed the side‐chain crystallization, leading to the disappearance of the side‐chain melting.

**FIGURE 2:**
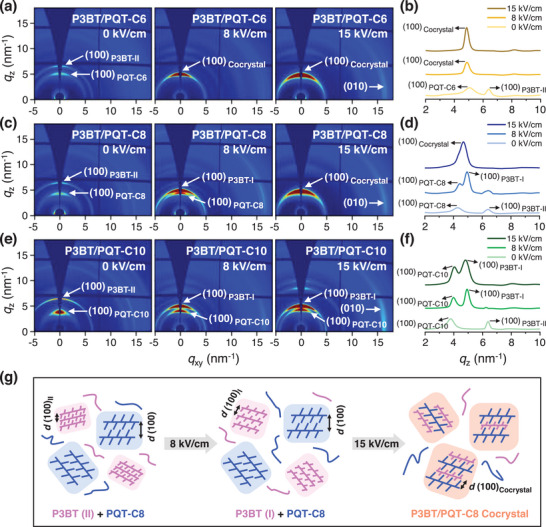
(a,c,e) 2D‐GIWAXS and (b,d,f) their corresponding 1D‐GIWAXS images of three blends: (a,b) P3BT/PQT‐C6, (c,d) P3BT/PQT‐C8, and (e,f) P3BT/PQT‐C10 produced from TCB under different EEF strengths (0, 8, and 15 kV/cm). (g) Schematic of the crystalline structure evolution in the representative P3BT/PQT‐C8 blend during the EEF process. At 0 kV/cm, P3BT in polymorph II (pink domain) and PQT‐C8 (blue domain) phase‐separate from each other and form respective crystal domains (left panel). P3BT transforms into polymorph I and phase‐separates with PQT‐C8 at 8 kV/cm (middle panel), but they form cocrystals (orange domain) at 15 kV/cm, in which their side chains interdigitate and form a single *d*_100_ (right panel).

**FIGURE 3:**
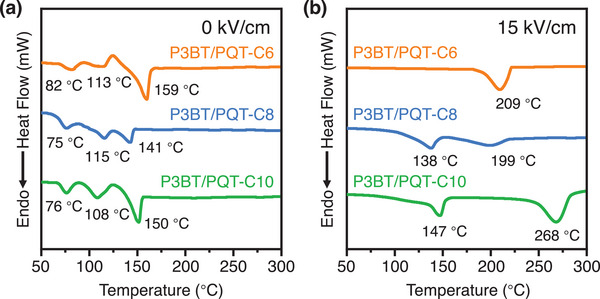
(a,b) DSC curves of three P3BT/PQT blends (P3BT/PQT‐C6, P3BT/PQT‐C8, and P3BT/ PQT‐C10) produced at (a) *E* = 0 kV/cm and (b) *E* = 15 kV/cm, respectively.

Subsequently, the P3BT/PQT blended films (i.e., P3BT/PQT‐C6, P3BT/PQT‐C8, and P3BT/PQT‐C10) under various *E* were elucidated by 2D‐GIWAXS. As for the P3BT/PQT‐C6 blended film without *E* (*E* = 0 kV/cm), phase separation between P3BT in polymorph II (*d*_100_ = 0.97 nm) and PQT‐C6 (*d*_100_ = 1.25 nm) was observed, as evidenced by their respective (100) peaks along the *q*_z_ direction (Figure 2a,b). At *E* of 8 and 15 kV/cm, a single (100) diffraction peak is observed at *q*_z_ = 4.9 nm^−1^ (*d*_100_ = 1.28 nm), with increased intensity at 15 kV/cm (Figure 2a,b). Due to the P3BT polymorph II‐to‐I change triggered by EEF, the P3BT in polymorph I had a larger *d*_100_ of 1.21 nm, and this *d*_100_ value was close to that of PQT‐C6 (*d*_100_ = 1.25 nm). Given the similar chemical structures and close *d*_100_ values of P3BT polymorph I and PQT‐C6, the newly formed single (100) peak at *q*_z_ = 4.9 nm^−1^ was attributed to the (100) peak of the P3BT/PQT‐C6 cocrystals. The DSC data further proved the formed P3BT/PQT‐C6 cocrystals by EEF. Three melting peaks at 82°C, 113°C, and 159°C appeared in the P3BT/PQT‐C6 blend at 0 kV/cm, which corresponded to the melting points of the PQT‐C6 side chains, P3BT polymorph II, and PQT‐C6 main chains, respectively (Figure 3). At *E* = 15 kV/cm, only a single endothermic peak of 209°C appeared, attributed to the cocrystal of P3BT polymorph I (268°C) and PQT‐C6 (155°C for its main chains). The DSC results corroborated the GIWAXS results, proving the phase separation and cocrystallization in P3BT/PQT‐C6 blend before and after EEF, respectively. The EEF first changed the P3BT polymorph II to I. Afterward, it cocrystallized with PQT‐C6 in which their different alkyl side chains interdigitated with each other (Figure S7a).

Similarly, the P3BT/PQT‐C8 blend showed a phase‐separated structure between P3BT polymorph II and PQT‐C8 at *E* = 0 kV/cm, characterized by two distinct (100) diffraction peaks at *q*_z_ = 6.4 nm^−1^ (*d*_100_ = 0.98 nm) and *q*_z_ = 4.3 nm^−1^ (*d*_100_ = 1.47 nm), respectively (Figure 2c,d). Different from the P3BT/PQT‐C6 blend, the P3BT/PQT‐C8 blend retained phase separation between P3BT polymorph I (*d*_100_ = 1.28 nm) and PQT‐C8 (*d*_100_ = 1.43 nm) at *E* = 8 kV/cm, while transformed to their cocrystals (*q*_z_ = 4.7 nm^−1^, *d*_100_ = 1.34 nm) at *E* = 15 kV/cm (Figure 2g). This suggested that the P3BT/PQT‐C8 blend required a higher *E* to form the cocrystals than the P3BT/PQT‐C6 blend. Moreover, the P3BT/PQT‐C8 cocrystals formed at 15 kV/cm showed two endothermic peaks at 138°C and 199°C, attributed to the melting of the PQT‐C8 main chains and P3BT/PQT‐C8 cocrystals, respectively (Figure 3). For comparison, P3BT/PQT‐C8 cocrystals with a lower content of PQT‐C8 (P3BT:PQT‐C8 = 1:0.5) formed at 15 kV/cm were treated by DSC as well, which still showed two endothermic peaks (128°C and 193°C) (Figure S8a). It proved that the appearance of two peaks in P3BT/PQT‐C8 cocrystals was due to the instability of the cocrystals upon heating, instead of an excess of PQT‐C8. To further prove it, the P3BT/PQT‐C8 cocrystal film was thermal annealed at 130°C (close to the PQT‐C8 backbone melting) for 20 min. In addition to the P3BT/PQT‐C8 cocrystal peak, a new diffraction peak, attributed to the (100) peak of PQT‐C8, appeared (Figure S8b). It proved that the P3BT/PQT‐C8 cocrystal was partially destroyed after heating and thus showed two melting peaks during DSC measurement. It also reflected a weaker cocrystalline ability of P3BT/PQT‐C8 than P3BT/PQT‐C6, in which the latter could maintain cocrystals and exhibit one endothermic peak throughout the DSC heating process.

As for the third P3BT/PQT‐C10 blend, the P3BT in the blend experienced the polymorph II to I transition as well at 8 or 15 kV/cm (Figure 2e,f). Nevertheless, as evidenced by the GIWAXS results (Figure 2e,f) and DSC data (Figure 3), P3BT and PQT‐C10 remained phase separated in the as‐cast state (*E* = 0 kV/cm) and throughout the EEF process (*E* = 8 and 15 kV/cm) (Figure S7b).

In the above discussion, the merging of (100) diffraction peaks proved the formation of cocrystals between P3BT and PQTs. In addition, their (010) diffraction peaks were analyzed as well. For three P3BT/PQT blended films (i.e., P3BT/PQT‐C6, P3BT/PQT‐C8, and P3BT/PQT‐C10), their (010) peak at 0 kV/cm was not clearly observed due to lower film crystallinity. At 15 kV/cm, their (010) peak was seen at *q*_x,y_ = 16.6 nm^−1^ (*d*_010_ = 0.38 nm) (Table S2) due to increased film crystallinity. For comparison, the P3BT homopolymer at 15 kV/cm had the (010) peak at *q*_x,y_ = 16.6 nm^−1^ (*d*_010_ = 0.38 nm). All three PQTs homopolymers (PQT‐C6, PQT‐C8, and PQT‐C10) showed the (010) peak at *q*_x,y_ = 16.7 nm^−1^ (*d*_010_ = 0.37 nm) at 15 kV/cm. Because the (010) peak positions of P3BT and three PQTs were very close, the (010) peak of their cocrystals did not exhibit distinct stacking distances compared with the two respective homopolymers. According to the pole figures, the fractions of edge‐on crystals in three P3BT/PQT blended films at 0 and 15 kV/cm were 73.6 %–91.2 % and 81.4 %–94.9 %, respectively (Table S3).

**FIGURE 4:**
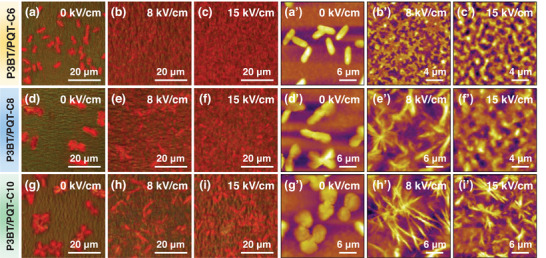
(a‐i) CPOM and (a’‐i’) the corresponding AFM images of three blends: (a‐c, a’‐c’) P3BT/PQT‐C6, (d‐f, d’‐f’) P3BT/PQT‐C8, and (g‐i, g’‐i’) P3BT/PQT‐C10 at different EEF strengths (0, 8, and 15 kV/cm).

The film morphology evolution of different P3BT/PQT blends and the corresponding homopolymers upon EEF was characterized by cross‐polarized optical microscopy (CPOM) and atomic force microscopy (AFM) (Figures 4; S9 and S10). The P3BT/PQT‐C6 blend at *E* = 0 kV/cm showed a short rod‐like morphology and a vague morphology without obvious morphology in the matrix (Figure 4a,a’), equivalent to the morphology of P3BT polymorph II and PQT‐C6, respectively. Compared to the complete spherulitic morphology of P3BT polymorph II without blending (Figure S9a), the P3BT morphology in the blend was affected by the PQT‐C6 and thus became underdeveloped. At *E* of 8 and 15 kV/cm, the morphology of the P3BT/PQT‐C6 blend became uniform and homogeneous by CPOM (Figure 4b,c). From a larger magnification by AFM, the blend displayed a star‐like and worm‐like morphology at *E* of 8 and 15 kV/cm, respectively (Figure 4b′,c′). Their film morphology on a larger scale changed from distinct morphologies between P3BT and PQT‐C6 at *E* = 0 kV/cm to homogeneous at 8 and 15 kV/cm, corroborating the phase‐separated structure and cocrystal structure detected by 2D‐GIWAXS on a smaller scale, respectively.

Similar to the P3BT/PQT‐C6 blend, the P3BT/PQT‐C8 and P3BT/PQT‐C10 blends exhibit two different features at *E* = 0 kV/cm, indicative of their phase‐separated structure (Figure 4d,d’,g,g’). Notably, the P3BT in the P3BT/PQT‐C10 blend showed a more circular and complete spherulitic morphology (Figure 4g,g’) than that in the other two blends (Figure 4a,a’,d,d’). Such spherulitic morphology is most likely P3BT homopolymer in polymorph II, indicating the highest phase‐separated degree between P3BT and PQT‐C10 among the three blends. At *E* = 8 kV/cm, the P3BT spherulites in the P3BT/PQT‐C8 and P3BT/PQT‐C10 blends changed into needle‐like morphology, characteristic of P3BT in polymorph I (Figure 4e,e’,h,h’). Thus, the P3BT morphology changed, accompanied by its polymorph II‐to‐I transformation. Due to stronger phase separation in P3BT/PQT‐C10 than in P3BT/PQT‐C8, the P3BT needles were larger and more obvious in the former. At *E* = 15 kV/cm, a more uniform and nodular morphology appeared in the P3BT/PQT‐C8 blend, corroborating their cocrystal structures by 2D‐GIWAXS (Figure 4f,f’). In contrast, the P3BT/PQT‐C10 blend retained the needle‐like morphology, in concert with their phase‐separated structure, even at *E* = 15 kV/cm (Figure 4i,i’).

### 2.2 Aggregation of P3BT/PQT Blends in the Solution Rendered by External Electric Field (EEF)

It is known that the solution aggregates of conjugated polymers have a great impact on the crystalline structures in the thin film [19, 20, 21, 22]. After thoroughly examining the polymorphs and morphologies of three P3BT/PQT blends in the thin film, it is crucial to go back to their solution state to reveal how these blends aggregated in solutions and connect these solution aggregates with their thin‐film polymorphs.

**FIGURE 5:**
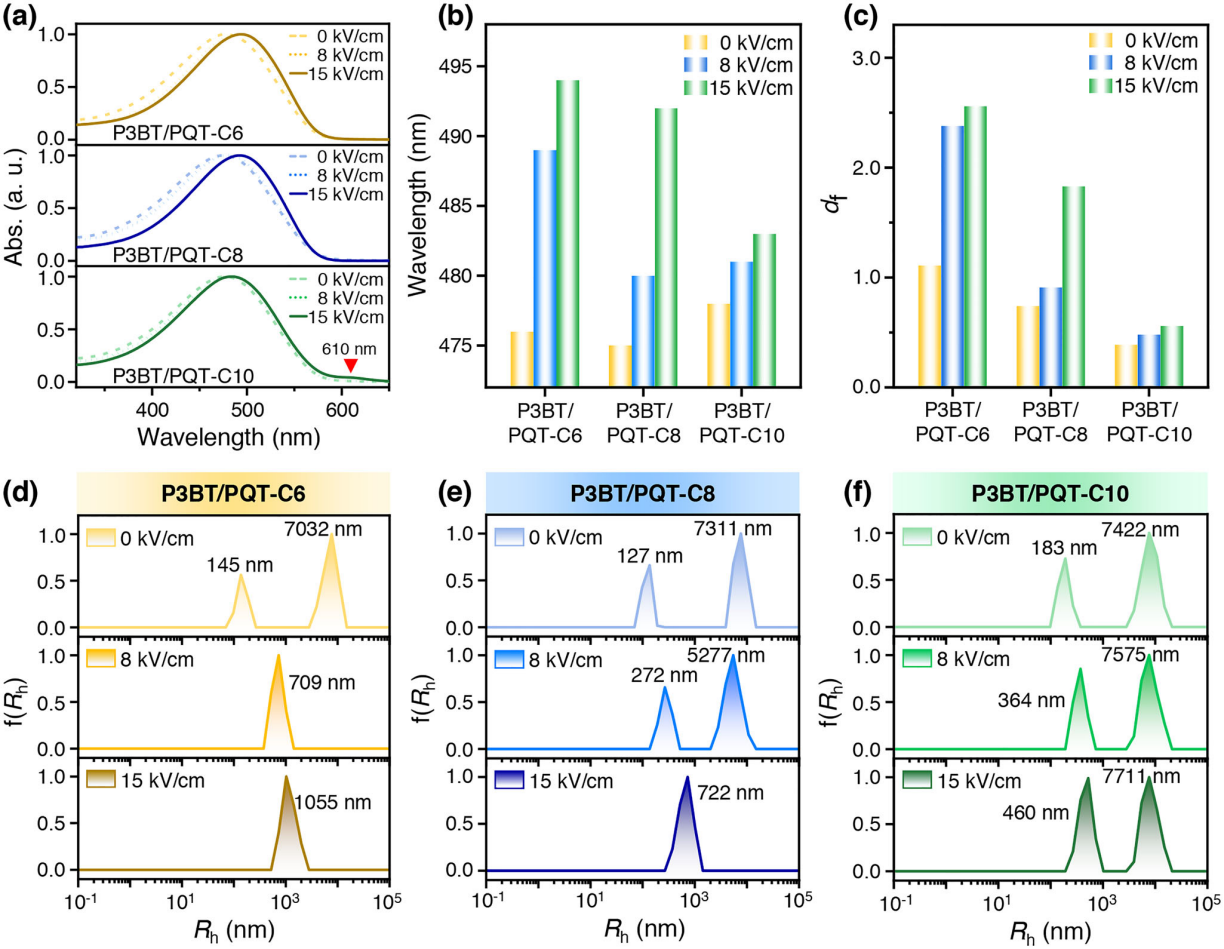
(a) UV–vis absorption spectra and (b) summary of the wavelength of the 0–2 transition peak of three P3BT/PQT blends (P3BT/PQT‐C6, P3BT/PQT‐C8, and P3BT/PQT‐C10) in the solution under different EEF strengths. (c) Summary of the fractal dimension (*d*_f_) values of three P3BT/PQT blends in the solution under different EEF strengths. (d–f) Hydrodynamic radius distributions (*R*_h_) of (d) P3BT/PQT‐C6, (e) P3BT/PQT‐C8, and (f) P3BT/PQT‐C10 in the solution under different EEF strengths.

First, three P3BT/PQT blended solutions and the corresponding homopolymers under various *E* were characterized by UV–vis spectroscopy (Figures 5; S11). The P3BT/PQT‐C6 blend exhibited a single absorption peak of 0–2 transition at 476 nm when *E* = 0 kV/cm (Figure 5a), which was located between that of P3BT homopolymer in polymorph II (459 nm) and PQT‐C6 homopolymer (482 nm) (Figure S11). This deviation from their respective absorption peak indicated the interaction between P3BT and PQT‐C6. The redshifted 0–2 transition of PQT‐C6 compared to P3BT indicated that PQT‐C6 had a more planar backbone conformation, which agreed well with DFT calculation (Figure S4). This 0–2 transition of the P3BT/PQT‐C6 blend showed a significant redshift (13 nm) to 489 nm at *E* = 8 kV/cm, and further redshifted (5 nm) to 494 nm at 15 kV/cm (Figure 5b). It implied the increased conjugation length and aggregation of P3BT/PQT‐C6 blend with the increased *E*.

Similarly, the P3BT/PQT‐C8 and P3BT/PQT‐C10 blends without EEF showed a 0–2 transition at 475 and 478 nm (Figure 5a), positioned between that of P3BT (459 nm) and PQT‐C8 (484 nm), and that of P3BT (459 nm) and PQT‐C10 (490 nm), respectively (Figure S11). The interaction between two components within each blend was proved by the deviation of the absorption peaks from their respective component. The 0–2 transition of the P3BT/PQT‐C8 and P3BT/PQT‐C10 redshifted to 480 and 481 nm at 8 kV/cm, and further to 492 and 483 nm at 15 kV/cm, respectively (Figure 5a,b). The redshift of the absorption peak proved the intensified aggregation of the two blends induced by EEF. Notably, although redshift of the 0–2 transition happened in all blended solutions upon EEF, the redshift degree was much larger in the system when the correspondent film occurred the transition from phase separation to cocrystallization, as reflected from P3BT/PQT‐C6 from 0 to 8 kV/cm (13 nm) and P3BT/PQT‐C8 from 8 to 15 kV/cm (12 nm) (Figure 5b). This demonstrates that these two blends exhibit pronounced response to the electric field at the point, contributing to their co‐crystallization in the film state. As for the P3BT/PQT‐C10 blend, it had the smallest redshift of the 0–2 transition (2–3 nm) and did not show the transition from phase separation to cocrystallization throughout the EEF process. Moreover, the P3BT/PQT‐C10 blend exhibited an absorption peak at 610 nm at 15 kV/cm (Figure 5a), corresponding to the 0–0 absorption peak of P3BT [37] (Figure S11). This peak was not observed in the other blends. It indicated P3BT and PQT‐C10 had the weakest interaction among the three blends, in which P3BT tended to form its own aggregates and thus exhibited its own characteristic peak. Briefly, the UV–vis results indicated that the EEF intensified the aggregation of all three P3BT/PQT blends in the solution, and the redshift degree was much larger when the corresponding blended film changed from phase separation between P3BT and PQT to their cocrystallization.

To further quantify the aggregates in the P3BT/PQT blended solutions, dynamic/static light scattering (DLS/SLS) was examined. DLS measures the hydrodynamic radius (*R*_h_) of various aggregates, reflecting the aggregate size (Figure 5d–f). The P3BT/PQT‐C6 blend showed a bimodal distribution of *R*_h_ at 145 and 7032 nm at *E* = 0 kV/cm (Figure 5d). The *R*_h_ distribution became unimodal upon EEF, at 709 and 1055 nm at 8 and 15 kV/cm, respectively. It meant the existence of at least two major different sizes of aggregates in the solution before EEF, but co‐aggregated as a whole after EEF. The bimodal‐to‐unimodal distributed *R*_h_ also reflected the strengthened interactions between P3BT and PQT‐C6. The changes in *R*_h_ correlated well with their thin‐film structures; that is, the bimodal distribution of *R*_h_ corresponded to the phase separation between P3BT and PQT‐C6 at 0 kV/cm, and the unimodal distribution corresponded to their cocrystallization at 8 and 15 kV/cm. Moreover, the increased E from 8 to 15 kV/cm significantly improved the co‐aggregation between P3BT and PQT‐C6 to form larger aggregates (from 709 to 1055 nm).

Such correlation was observed in the P3BT/PQT‐C8 and P3BT/PQT‐C10 blends as well. For the P3BT/PQT‐C8 blend, it had a bimodal distribution of *R*_h_ at 0 (127 and 7311 nm) and 8 kV/cm (272 and 5277 nm), in which the size of the two types of main aggregates got closer to each other upon EEF (Figure 5e). Afterward, the two *R*_h_ values became one at 722 nm at 15 kV/cm, demonstrating the co‐aggregation between P3BT and PQT‐C8 and thus strengthened intermolecular interaction between them (Figure 5e). It aligned well with the phase‐separated structures in thin films of P3BT/PQT‐C8 at 0 and 8 kV/cm, while cocrystalline structures at 15 kV/cm (Figure 2c). In the case of the P3BT/PQT‐C10 blend, a bimodal distribution of *R*_h_ was retained throughout the whole process, accompanied by the increased *R*_h_ with the increased *E* (Figure 5f). This suggested relatively weaker intermolecular interactions between P3BT and PQT‐C10 during the EEF, which were insufficient to induce their co‐aggregation even under high EEF strength. It corresponded well to their phase‐separated structures in thin films always (Figure 2e).

SLS was further used to study the compactness of different P3BT/PQT aggregates by measuring their fractal dimension (*d_f_*) (see the detailed calculation in the Supporting Information) (Figure S12). It reflects the chain packing density in the aggregates, and a larger *d_f_* indicates a more compact chain packing [38]. Before applying the EEF, the *d_f_* values of three P3BT/PQT blends were 1.11, 0.74, and 0.39 for P3BT/PQT‐C6, P3BT/PQT‐C8, and P3BT/PQT‐C10, respectively (Figure 5c). It meant that with the increased alkyl side chains of PQTs, the blended aggregates became more and more looser due to gradually weakened intermolecular interactions between P3BT and PQTs, among which P3BT/PQT‐C6 had the most compact aggregates. Upon the EEF, all three P3BT/PQT blends had increased *d*_f_ with the increased *E* (Figure 5c). It indicated these aggregates became compact with strengthened intermolecular interaction induced by EEF, regardless of whether the *R*_h_ distribution of aggregates in the solution was bimodal or unimodal.

Interestingly, it was found that the increment of *d*_f_ was larger when the distribution of *R*_h_ transitioned from bimodal to unimodal. Specifically, the *d*_f_ of P3BT/PQT‐C6 increased 114 % to 2.38 from 0 to 8 kV/cm when their *R*_h_ changed from bimodal to unimodal at this stage, much larger than 8 % increment from 8 to 15 kV/cm with the retention of unimodal *R*_h_ during this process. Similarly, the *d*_f_ of P3BT/PQT‐C8 increased 101 % to 1.83 from 8 to 15 kV/cm, much larger than 23 % increment from 0 to 8 kV/cm. In contrast, a slight and more uniform increment of *d*_f_ (13 %∼23 %) was observed in P3BT/PQT‐C10 during the EEF process, in which a bimodal distribution of *R*_h_ remained throughout the whole process. The trend of *d*_f_ increment agreed well with the trend of the redshift of the 0–2 transition in UV–vis results (Figure 5b). These differences suggested that the intermolecular interactions of P3BT/PQT aggregates were particularly strengthened during the transition of *R*_h_ from bimodal to unimodal distribution. It is noted that when characterizing the solutions by UV–vis and DLS/SLS, the solutions were diluted to 1 mg/mL in order not to exceed the measurement range of the instruments. Since different P3BT/PQT blended solutions (i.e., P3BT/PQT‐C6, P3BT/PQT‐C8, and P3BT/PQT‐C10) were measured at the same concentration (1 mg/mL), their aggregation behavior can be compared. Moreover, these three P3BT/PQT blended solutions at 1 mg/mL were also exposed to the EEF; the formed films showed similar structural evolution trends with a weaker diffraction signal (Figure S13). It proved this concentration variation did not influence the solution‐to‐film structural evolution.

### 2.3 Formation Mechanism of Cocrystalline and Phase‐Separated Structures in P3BT/PQT Blended Films by External Electric Field (EEF)

After thoroughly analyzing the crystalline structures and morphologies of different P3BT/PQT blends in thin films and their corresponding aggregates in solutions, we focus our attention on establishing the connection between the solution and film states. The above results demonstrated that the molecular structure (side‐chain length of PQTs) and EEF strength *E* effectively tuned the aggregation degree of three P3BT/PQT blends in the solution, thereby strongly influencing the cocrystalline and phase‐separated structures in the thin film. Specifically, increasing the alkyl side‐chain length of PQTs facilitated the solubility in the solution, thereby weakening the aggregation of PQTs and the aggregation between P3BT and PQTs. Meanwhile, the dipole moment modulated by the EEF also changed intermolecular interactions, thereby affecting the solution aggregation. The relationship between the dipole moment and the EEF strength *E* is specifically manifested as: [26, 39]

(1) $$p=\alpha \left(E+E_{induced}\right)\;$$

where *E*_induced_ is the induced EEF strength by other molecules or atoms, and *α* is the polarizability tensor of molecules, which describes the magnitude and direction of the changed dipole moment. Thus, the molecular dipole moment is proportional to the EEF strength, and a larger EEF strength leads to an enhanced dipole–dipole interaction between molecules.

When the P3BT/PQT blended solution was not exposed to EEF (*E* = 0 kV/cm), the intermolecular interactions between molecules were relatively weak without the directional guidance from the EEF. P3BT and PQTs preferentially formed their own aggregates instead of their co‐aggregation, as evidenced by their bimodal distribution of *R*_h_ from DLS (Figure 5d–f) and the loosely packed structures from SLS (Figures 5c; S12). Consequently, during the solvent evaporation and film‐forming process, P3BT and PQTs tended to retain their own aggregates and thus crystallized separately, ultimately forming phase‐separated structures between P3BT and PQTs. Notably, the phase‐separated degree between P3BT and PQTs progressively increased with the extended side chains of PQTs, in which P3BT/PQT‐C10 had the strongest phase‐separated degree. It can be reflected from the characteristic 0–0 absorption peak (610 nm) of P3BT appeared in the UV–vis (Figure S11a) and the smallest *d*_f_ value (0.39) among three P3BT/PQT blends in the solution (Figure S12c), and the most complete spherulitic morphology of P3BT in the three P3BT/PQT blends in the films (Figure 4g,g’). Because the longest alkyl side chains of PQT‐C10 reduced its aggregation capability, and simultaneously weakened the intermolecular interactions between P3BT and PQT‐C10. Therefore, the weakest co‐aggregation between P3BT and PQT‐C10 in the solution and the strongest phase‐separated degree in the film were observed in this blend. Notably, the molecular weight of PQT‐C10 (*M*_n_ = 20.6 kg/mol) was larger than that of the other two PQTs. To exclude the possibility that longer PQT‐C10 chains may interfere with the cocrystal formation with P3BT, another PQT‐C10 with a lower molecular weight (*M*_n_ = 11.1 kg/mol) was also used and blended with P3BT, which remained phase‐separated structures during the EEF process (Figure S14). This again demonstrated that the P3BT/PQT‐C10 blend exhibited the strongest phase‐separated degree in the film.

When these P3BT/PQT blended solutions were exposed to the EEF, the π‐electron clouds in the backbone chains of P3BT and PQT were polarized and rearranged, leading to aligned dipole moments along the EEF direction and strengthened dipole–dipole interactions. Therefore, the solution aggregates of all three P3BT/PQT blends became denser, and this was proved by the redshift of the 0–2 transition peak in UV–vis spectra (Figure 5a) and increased *d*_f_ revealed by SLS (Figure 5c). As for each P3BT/PQT blend, its aggregation behavior was influenced by the alkyl side chains of PQT and the EEF strength.

**FIGURE 6:**
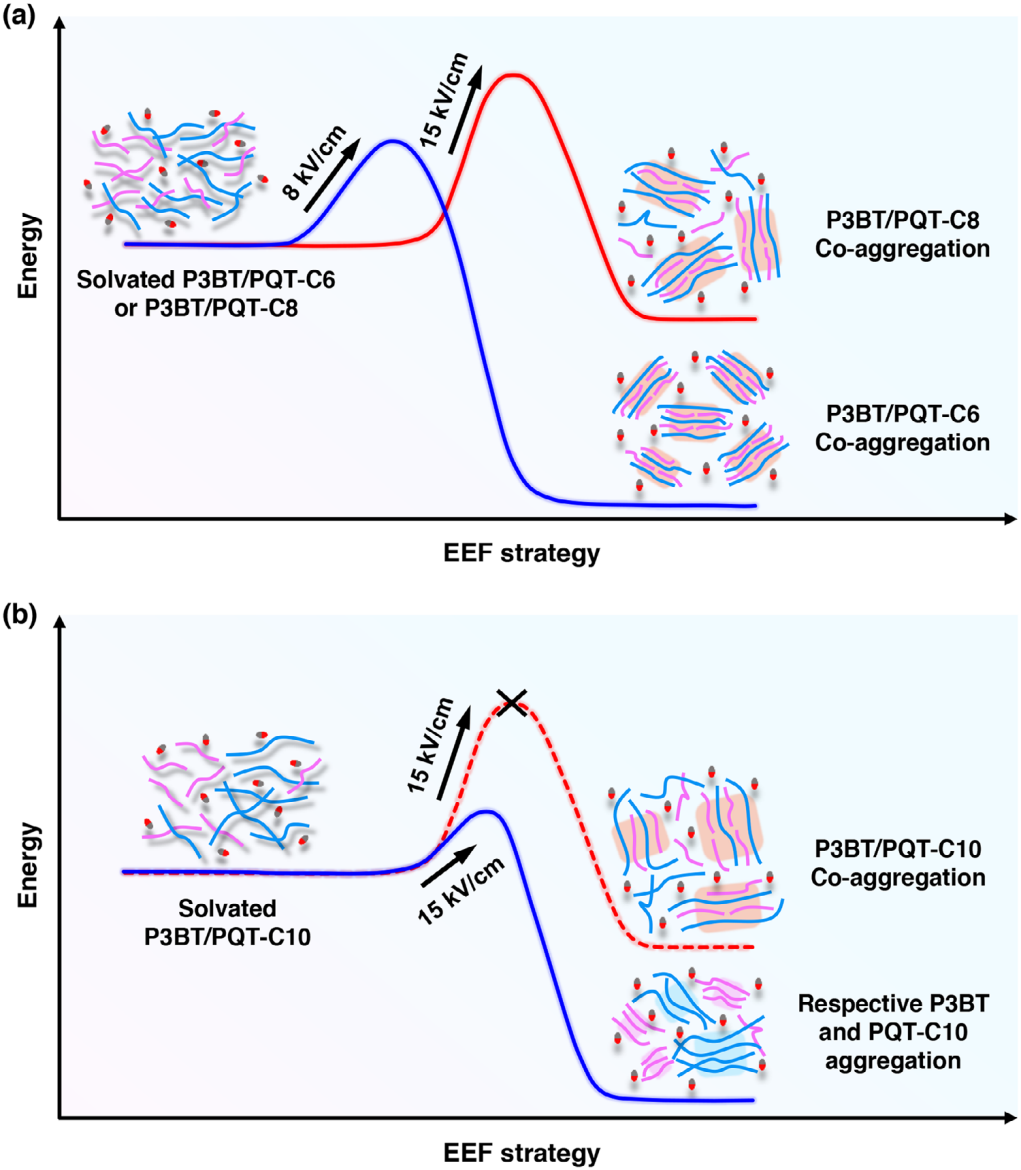
Schematic illustration of the potential energy of different P3BT/PQT blends from solvated dispersed chains to different solution aggregates under different EEF strengths. (a) The EEF strategy helped the P3BT/PQT‐C6 and P3BT/PQT‐C8 blends to overcome the energy barrier by enhancing intermolecular interactions, resulting in their co‐aggregation at 8 and 15 kV/cm, respectively. (b) The P3BT/PQT‐C10 blend failed to form co‐aggregates by the EEF strategy at 15 kV/cm due to weak intermolecular interactions, and formed respective P3BT and PQT‐C10 aggregates instead.

Specifically, for the P3BT/PQT‐C6 blend, the shortest alkyl side chains of PQT‐C6 exhibited the weakest solubilizing effect, resulting in the most rigid polymer backbone and the strongest chain aggregation capability. Consequently, dipole–dipole interactions were more readily generated under the EEF, leading to stronger intermolecular interactions. This effect facilitated the solvated P3BT/PQT‐C6 to overcome the energy barrier to form their co‐aggregates (Figure 6a). Therefore, the P3BT/PQT‐C6 blend could change from bimodal to unimodal distribution of *R*_h_ at low *E* of 8 kV/cm, and thus form cocrystals in the thin film at this *E*. In terms of P3BT/PQT‐C8, due to the reduced aggregation capability of PQT‐C8, it needed stronger dipole–dipole interactions to overcome a higher energy barrier of co‐aggregates (Figure 6a). As a result, this blend retained a bimodal distribution of *R*_h_ at 8 kV/cm, but only transitioned to a unimodal distribution at 15 kV/cm. Accordingly, it underwent the transformation from phase separation to cocrystallization in the thin film at a higher 15 kV/cm.

In contrast, the solvated P3BT/PQT‐C10 failed to form effective co‐aggregation even at 15 kV/cm due to the pronounced solubilizing effect coming from the longest side chains of PQT‐C10, which significantly reduced the intermolecular attractive forces, thereby preventing the blend from overcoming the needed highest energy barrier for co‐aggregation (Figure 6b). This led to a bimodal distribution of *R*_h_ in the solution and a phase‐separated structure in the thin film throughout the EEF process.

### 2.4 Charge Transport Properties of P3BT/PQT Blended Films with Cocrystalline and Phase‐Separated Structures

**FIGURE 7:**
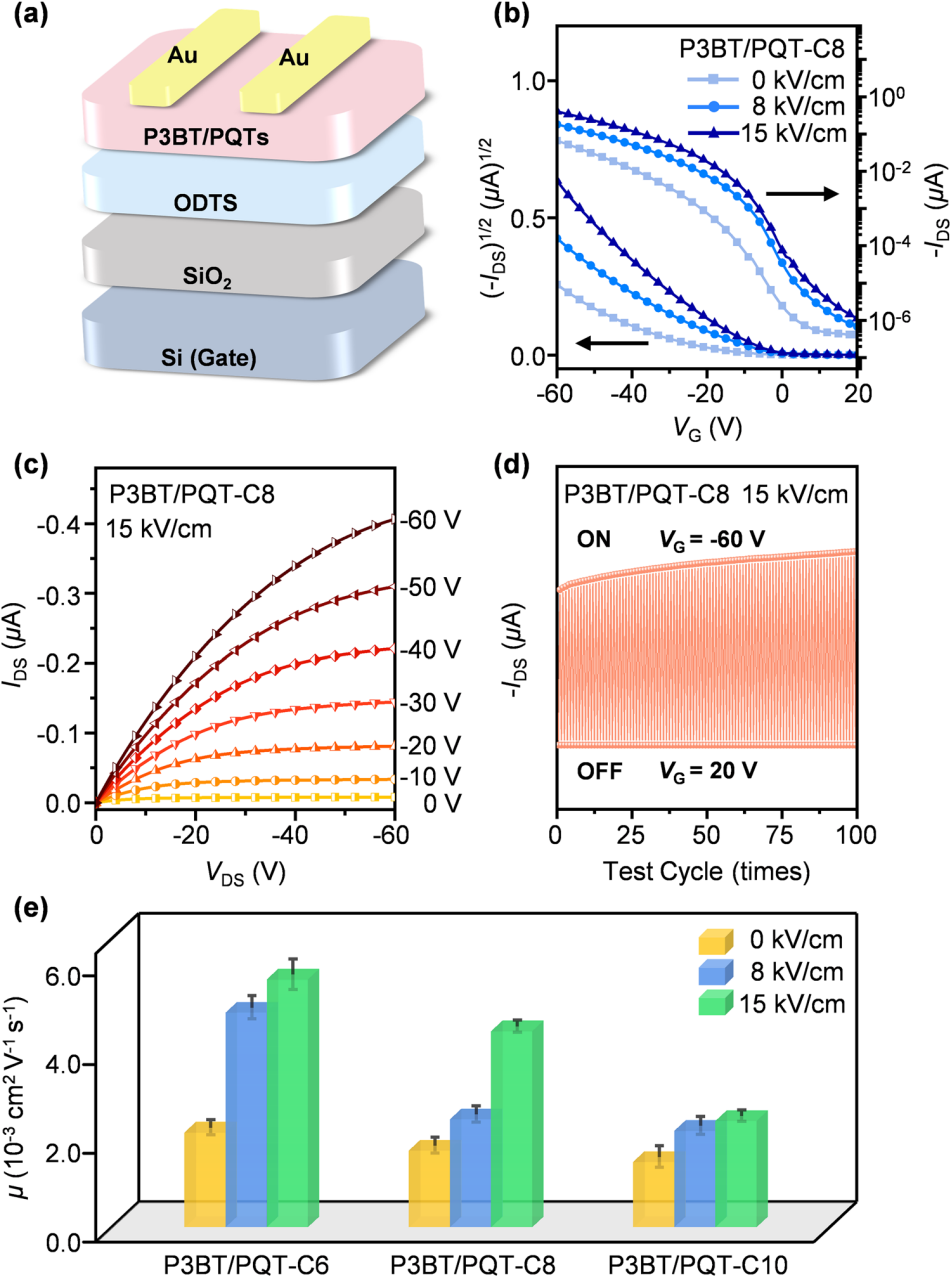
(a) Schematic illustration of the OFET device configuration based on P3BT/PQT blends. (b) Representative transfer and (c) output curves of the P3BT/PQT‐C8 blended films formed at various EEF strengths. (d) Cycle stability of a typical OFET prepared from P3BT/PQT‐C8 blended film under 15 kV/cm with continuous ON (*V*_G_ = −60 V) and OFF (*V*_G_ = 20 V) cycles for 100 times. (e) Average charge mobility of three blends (P3BT/PQT‐C6, P3BT/PQT‐C8, and P3BT/PQT‐C10) prepared at various EEF strengths.

Finally, the correlation between various cocrystalline and phase‐separated structures of P3BT/PQT blends and carrier transport properties was built up based on their thin films as the OFET active layers (Figure 7a). As an example, the transfer and output curves of the P3BT/PQT‐C8 blend under various conditions were shown in Figure 7b,c, and two other blends (P3BT/PQT‐C6, P3BT/PQT‐C10) as well as single homopolymers (P3BT, PQT‐C6, PQT‐C8, and PQT‐C10) were given in Figures S15–S18. The charge carrier mobility (*µ*) of these devices was summarized in Figure 7e and Table S4. As for single homopolymer systems, the average *µ* was 6.25 × 10^−4^, 1.03 × 10^−3^, 1.44 × 10^−3^, and 1.60 × 10^−3^ cm^2^ V^−1^ s^−1^ for P3BT, PQT‐C6, PQT‐C8, and PQT‐C10 at *E* = 0 kV/cm, respectively (Table S4).

In the case of three P3BT/PQT blends, their average *µ* was 2.13 × 10^−^^3^ cm^2^ V^−1^ s^−1^, 1.73 × 10^−^^3^ cm^2^ V^−1^ s^−1^, and 1.47 × 10^−^^3^ cm^2^ V^−1^ s^−1^ at *E* = 0 kV/cm for P3BT/PQT‐C6, P3BT/PQT‐C8, and P3BT/PQT‐C10, respectively (Figure 7e; Table S4). Compared with the individual polymers, the *µ* of P3BT/PQT‐C6 and P3BT/PQT‐C8 blends were higher than those of respective components, while the P3BT/PQT‐C10 blend had the *µ* between P3BT and PQT‐C10. All three P3BT/PQT blends displayed increased *µ* with the increased *E*, among which P3BT/PQT‐C6 had the highest *µ* of 6.07 × 10^−^^3^ cm^2^ V^−1^ s^−1^ at *E* = 15 kV/cm. Further scrutiny shows that the increment of *µ* was more significant at the transition point from phase‐separated to cocrystalline structures. Specifically, the *µ* of P3BT/PQT‐C6 increased 127 % to 4.84 × 10^−^^3^ cm^2^ V^−1^ s^−1^ from 0 to 8 kV/cm, much larger than 15 % increment from 8 to 15 kV/cm, aligned well with the trend that the *µ* of P3BT/PQT‐C8 increased 82 % to 4.42 × 10^−^^3^ cm^2^ V^−1^ s^−1^ from 8 to 15 kV/cm, much larger than 40 % increment from 0 to 8 kV/cm. In contrast, for the P3BT/PQT‐C10 blend with phase‐separated structures before and after EEF, their *µ* showed a slight or moderate increment (11 %∼47 %) from 0 to 8 and up to 15 kV/cm. Exemplified by P3BT/PQT‐C8 blend at *E* = 15 kV/cm, the device demonstrated excellent operational stability over 100 consecutive switching cycles (Figure 7d).

Different OFET performances of P3BT/PQT blends were closely related to their crystalline structures in thin films. Before EEF, a comprehensive analysis of GIWAXS, CPOM, and AFM revealed that these three blends exhibited an increased phase‐separated degree between P3BT and PQT with an extended side‐chain length of PQT. The P3BT/PQT‐C10 had the highest phase‐separated degree among the three blends, as evidenced by the most circular and complete spherulitic morphology of P3BT among the three blends, resembling the morphology of P3BT homopolymer the most. Because the intensified phase separation between P3BT and PQT increased the crystal grain boundary and made it sharper, disfavoring the charge transport. Therefore, these three blends had decreased *µ* with extended alkyl side chains, among which P3BT/PQT‐C10 had the lowest average *µ* of 1.47 × 10^−^^3^ cm^2^ V^−1^ s^−1^ at 0 kV/cm.

At *E* = 8 kV/cm, all three P3BT/PQT blends showed increased *µ* (2.13 × 10^−^^3^∼4.84 × 10^−^^3^) compared to their as‐cast state without EEF, among which the P3BT/PQT‐C6 blend showed a much larger increment (127 %) than the other two blends (40 %–47 %). The EEF‐improved *µ* was also manifested in single homopolymer systems (Table S4). Because the EEF promoted the film crystallinity, and it is reasonable that charge transport occurs more efficiently in crystalline regions than in amorphous regions [40], all three blends had higher *µ* than their as‐cast state at 0 kV/cm. The largest increment of *µ* found in the P3BT/PQT‐C6 blend was because it transformed into cocrystalline structures at 8 kV/cm, whereas the other two blends retained phase separation under the same condition. It underpins the cocrystal‐facilitated charge transport due to the synergy of two components, since the higher planarity of PQT‐C6 backbones facilitated intrachain charge transport and the stronger *π–π* stacking of P3BT favored interchain charge transport.

Increasing the *E* to 15 kV/cm further increased the *µ* of all three P3BT/PQT blends. The film crystallinity of the three blends was further increased, which was favorable for the charge transport. Among them, the P3BT/PQT‐C8 showed the largest increment of *µ* (82 %) from 8 to 15 kV/cm, higher than the other two blends under the same condition (11 %–15 %). Because the P3BT/PQT‐C8 blend changed from phase separation between P3BT and PQT‐C8 to their cocrystals at this *E*, resulting in significantly improved *µ*. It was similar to the case of P3BT/PQT‐C6, which showed a much increased *µ* (increment of 127 %) after the transition to cocrystals at 8 kV/cm. Moreover, the fractions of edge‐on crystals in three P3BT/PQT blended films at 0 and 15 kV/cm were 73.6 %–91.2 % and 81.4 %–94.9 %, respectively (Table S3). The slightly increased edge‐on crystals may also favor the charge transport. Besides, these P3BT/PQT blends had lower film roughness in the films with cocrystalline structures (15–38 nm) than those of phase‐separated structures (230–428 nm). The reduced film roughness in cocrystal films also contributed to the increased *µ*. However, the enhancement in charge carrier mobility was largely independent of film thickness, as evidenced by the close thickness (∼680 nm) of the P3BT/PQT‐C6 film at 15 kV/cm with the highest mobility and the P3BT/PQT‐C10 film at 0 kV/cm with the lowest mobility. Taken together, these results further substantiate the concept of cocrystal‐facilitated charge transport, that is, P3BT/PQT cocrysals favored charge transport in thin films rather than their phase‐separated structures.

## 3 Conclusion

In summary, we demonstrated the ability to produce the cocrystals in P3BT/PQT blends via a simple and robust EEF strategy and correlated their aggregation behaviors in the solution with cocrystalline structures in the film. All three blends (P3BT/PQT‐C6, P3BT/PQT‐C8, and P3BT/PQT‐C10) exhibited phase‐separated structures in their as‐cast state without EEF. Upon the application of EEF, a structural transition from phase separation to cocrystallization was successfully achieved in P3BT/PQT‐C6 and P3BT/PQT‐C8 blends at the EEF strengths of 8 and 15 kV/cm, respectively. In contrast, P3BT/PQT‐C10 retained its phase‐separated structure throughout the EEF process even under the highest EEF strength of 15 kV/cm. Tracing back the P3BT/PQT blended solutions demonstrated that the intrinsic (i.e., alkyl side‐chain length of PQTs) and extrinsic factors (i.e., EEF strength) collectively impacted the P3BT/PQT aggregates in the solution, thereby yielding various crystalline structures in the film. Without the EEF, P3BT and PQT preferentially aggregated and separated in the solution due to weak intermolecular interactions, resulting in phase‐separated structures in the film. When exposed to the EEF, the EEF helped P3BT/PQT‐C6 and P3BT/PQT‐C8 blends to overcome the energy barrier to form co‐aggregates in the solution, and thus formed cocrystals in the film. However, the pronounced solubilizing effect of the longest side chains of PQT‐C10 weakened intermolecular interactions and prevented the P3BT/PQT‐C10 blend from forming co‐aggregation even at 15 kV/cm, leading to a phase‐separated structure in the film throughout the EEF process. Different crystalline structures of P3BT/PQT blends greatly impacted the resulting device performance, where their cocrystalline structures displayed a much higher charge transport property than the corresponding phase‐separated structures. Clearly, this work demonstrated the effectiveness of the EEF strategy in constructing cocrystals in conjugated polymer blends and the concept of cocrystal‐facilitated charge transport. We envision that this approach can be extended to a broader range of conjugated polymer systems, offering a novel cocrystal engineering strategy for optimizing the performance of organic optoelectronic devices.

## Conflicts of Interest

The authors declare no conflicts of interest.

## Supporting information




**Supporting File**: advs73444‐sup‐0001‐SuppMat.pdf.

## Data Availability

The data that support the findings of this study are available from the corresponding author upon reasonable request.
