# Supplementary material for: Cocrystal Engineering of Conjugated Polymer Blends via External Electric Field for Enhanced Charge Transport
Source: Adv Sci (Weinh). 2026 Jan 7;13(13):e20457. doi: 10.1002/advs.202520457 (PMC12955856; doi:10.1002/advs.202520457)
Supplement: Supplementary file 1 — Supporting File: advs73444‐sup‐0001‐SuppMat.pdf. [file ADVS-13-e20457-s001.pdf]

Supporting Information

Cocrystal Engineering of Conjugated Polymer Blends via External Electric Field for Enhanced Charge Transport

*Yanan Guo, Hao Zheng, and Juan Peng**

State Key Laboratory of Molecular Engineering of Polymers, Department of Macromolecular Science, Fudan University, Shanghai 200438, China

Corresponding Author

E-mail: juanpeng@fudan.edu.cn (J. Peng)

The Supporting Information file includes:

1. Experimental Section

Materials.

Synthesis of Poly(3-butylthiophene) (P3BT).

Synthesis of Poly[3,3‴-bis(6-hexylthiophenyl)quaterthiophene] (PQT-C6).

Synthesis of Poly[3,3‴-bis(8-octylthiophenyl)quaterthiophene] (PQT-C8).

Synthesis of Poly[3,3‴-bis(10-decylthiophenyl)quaterthiophene] (PQT-C10).

Density Functional Theory (DFT) Calculation.

Preparation of P3BT/PQT Blended Solutions and Films via the External Electric Field (EEF) Strategy.

Calculation of Fractal Dimension (d_f) via Static Light Scattering (SLS).

Fabrication of Organic Field-Effect Transistors.

Statistical Analysis.

Characterization.

References.

2. Table S1-S4

Table S1. Summary of Molecular Weights and Polydispersity Index (PDI) of P3BT, PQT-C6, PQT-C8 and PQT-C10.

Table S2. Summary of 2D-GIWAXS Results for Four Homopolymers and Three P3BT/PQT Blended Films under Different Conditions.

Table S3. Summary of Molecular Orientation in Three P3BT/PQT Blended Films under External Electric Field (EEF).

Table S4. Summary of OFET Device Performances of Four Homopolymers and Three P3BT/PQT Blended Films Produced under Different Conditions.

3. Figure S1-S18

Figure S1. Synthesis routes of (a) P3BT and (b) PQTs (i.e., PQT-C6, PQT-C8, and PQT-C10) with different alkyl side chains.

Figure S2. GPC profiles of (a) P3BT (M_n = 3.7 kg/mol), (b) PQT-C6 (M_n = 15.8 kg/mol), (c) PQT-C8 (M_n = 18.9 kg/mol) and (d) PQT-C10 (M_n = 20.6 kg/mol).

Figure S3. ^{1}H NMR spectra of (a) P3BT, (b) PQT-C6 (c) PQT-C8 and (d) PQT-C10 in $CDCl_3$ (*****).

Figure S4. DFT-simulated geometries of the dodecamers of (a) P3BT and the trimers of (b) PQT-C6, (c) PQT-C8, (d) PQT-C10 in both top view (left) and side view (right).

Figure S5. Schematic representation of two-dimensional grazing incidence wide angle X-ray scattering (2D-GIWAXS) experiment on the conjugated polymer film.

Figure S6. (a, b) DSC curves of four homopolymers (P3BT, PQT-C6, PQT-C8, and PQT-C10) films produced at (a) E = 0 kV/cm and (b) E = 15 kV/cm, respectively.

Figure S7. (a) Schematic of the crystalline structure evolution in the P3BT/PQT-C6 blend during the EEF process. At 0 kV/cm, P3BT in polymorph II (pink domain) and PQT-C6 (yellow domain) phase-separate with each other and form respective crystal domains (left panel). P3BT transforms into polymorph I at 8 kV/cm (middle panel), and then form cocrystals (orange domain) at this EEF strength (8 kV/cm), in which their alkyl side chains interdigitate with each other and produce a single d_{100} (right panel). (b) Schematic of the crystalline structure evolution in the P3BT/PQT-C10 blend during the EEF process. At 0 kV/cm, P3BT in polymorph II (pink domain) and PQT-C10 (green domain) phase-separate with each other and form respective crystal domains (left panel). P3BT transforms into polymorph I and phase-separate with PQT-C10 at 8 kV/cm (middle panel). They remain phase separation when E is further increased to 15 kV/cm (right panel).

Figure S8. (a) DSC curves of P3BT/PQT-C8 (1:0.5) blend under the EEF strength of 15 kV/cm. (b) XRD profiles of P3BT/PQT-C8 (1:1) blend after thermal annealing at 130 °C.

Figure S9. (a-l) CPOM images of (a-c) P3BT, (d-f) PQT-C6, (g-i) PQT-C8, (j-l) PQT-C10 films produced under different EEF strengths.

Figure S10. The AFM topography images of three P3BT/PQT blended films: (a) P3BT/PQT-C6, (b) P3BT/PQT-C8, and (c) P3BT/PQT-C10 at different EEF strengths (0, 8, and 15 kV/cm).

Figure S11. (a-d) UV-vis absorption spectra of (a) P3BT, (b) PQT-C6, (c) PQT-C8, (d) PQT-C10 solutions under different EEF strengths.

Figure S12. Plots of log $I_{(q)}$ versus log q for (a) P3BT/PQT-C6, (b) P3BT/PQT-C8 and (c) P3BT/PQT-C10 blends in the solution at different EEF strengths. The fractal dimension (d_f) of the solution aggregates is obtained from the slop of the log $I_{(q)}$ versus log q after linear fitting.

Figure S13. XRD profiles of three P3BT/PQT blended films (1 mg/mL) produced under different EEF strengths (0, 8 and 15 kV/cm): (a) P3BT/PQT-C6, (b) P3BT/PQT-C8 and (c) P3BT/PQT-C10.

Figure S14. XRD profiles of P3BT/PQT-C10 blended films produced under different EEF strengths (0, 8 and 15 kV/cm), in which the PQT-C10 had a lower M_n of 11.1 kg/mol.

Figure S15. (a, c, e) Transfer and output (b, d, f) curves of P3BT/PQT-C6 blended films formed at various EEF strengths.

Figure S16. (a, b) Output curves of P3BT/PQT-C8 blended films formed at various EEF strengths.

Figure S17. (a, c, e) Transfer and output (b, d, f) curves of P3BT/PQT-C10 blended films formed at various EEF strengths.

Figure S18. (a, c, e, g, i, k, m, o) Transfer and (b, d, f, h, j, l, n, p) output curves of (a-d) P3BT, (e-h) PQT-C6, (i-l) PQT-C8, and (m-p) PQT-C10 films formed at various EEF strengths.

Experimental Section

Materials.

2-bromo-5-iodo-3-butylthiophene (BT) was synthesized based on the previous literatures.[1] 5,5′-Dibromo-4,4′-dihexyl-2,2′-bithiophene (2TC6-2Br), 5,5′-dibromo-4,4′-dioctyl-2,2′-bithiophene (2TC8-2Br), 5,5′-dibromo-4,4′-didecyl-2,2′-bithiophene (2TC10-2Br) and 5,5'-bis(trimethylstannyl)-2,2'-bithiophene (2T-2Sn) were purchased from Nanjing Zhiyan Technology Co., Ltd. Tris(dibenzylideneacetone)dipalladium (0) [$Pd_2(dba)_3$] and tri-(*o*-tolyl)phosphine [P(*o*-tol)$_3$] were purchased from Shanghai Macklin Biochemical Co., Ltd. The isopropylmagnesium chloride (*i*-PrMgCl) (2.0 M in tetrahydrofuran (THF)), [1,3-bis(diphenylphosphino)propane]-dichloronickel(II) (Ni(dppp)Cl_2), octadecyltrichlorosilane (ODTS) and anhydrous chlorobenzene (CB) were acquired from Sigma-Aldrich. THF was freshly dried over sodium and benzophenone. Other solvents and reagents were purchased from the Sinopharm Chemical Reagent Co., Ltd. All reagents were used as received.

Synthesis of Poly(3-butylthiophene) (P3BT).

P3BT (M_n = 3.7 kg/mol, PDI= 1.43) was synthesized via Kumada catalyst-transfer polycondensation (KCTP).[2] The monomer 2-bromo-5-iodo-3-butylthiophene (BT, 1.50 g, 4.35 mmol) was dissolved in THF (60 mL) and injected into a preheated and dried three-neck flask. The solution was cooled to 0 °C and isopropylmagnesium chloride (*i*-PrMgCl) (2.2 mL, 4.4 mmol) in THF was added and the mixture was stirred for 30 min under N_2 atmosphere. Afterwards the solution was heated to 35 °C, [1,3-bis(diphenylphosphino)propane]-dichloronickel(II) (Ni(dppp)Cl_2) catalyst (119.24 mg, 0.22 mmol) was added, and the obtained mixture was stirred at 35 °C for 3 h. After that, the reaction was quenched by the addition of HCl (aq) (50 wt %) and the product was deposited into methanol and hexane, and dried under vacuum to obtain dark brown solid. The polymer was purified using the Soxhlet extraction method, with methanol, acetone, n-hexane, and chloroform used in sequence to remove unreacted monomers and oligomers. Finally, the product was dissolved in chloroform and concentrated by rotary evaporation to obtain the final product.

Synthesis of Poly[3,3'''-bis(6-hexylthiophenyl)quaterthiophene] (PQT-C6).

PQT-C6 (M_n = 15.8 kg/mol, PDI= 2.34) was synthesized by Stille copolymerization according to previously method.[3,4] 2TC6-2Br (391.3 mg), 2T-2Sn (391.7 mg), $Pd_2(dba)_3$ (29.1 mg) and P(*o*-tol)$_3$ (38.7 mg) were added into a microwave reaction flask, dissolved in 15 mL anhydrous chlorobenzene (CB) and fully stirred under the action of magneton. The reaction

flask was continuously purged with nitrogen gas during the dissolution process to maintain an inert atmosphere. The flask was then placed in a microwave reactor, stirred for 1 min, reacted at 140 °C for 2 min, reacted at 160 °C for 2 min, and then reacted at 180 °C for 20 min. After the reaction, the solution was poured into a mixture containing 400 mL of methanol and 20 mL of hydrochloric acid, allowed to settle for 30 min, and then filtered to obtain the crude product. The polymer was purified using the Soxhlet extraction method, with methanol, acetone, n-hexane, and chloroform used in sequence to remove unreacted monomers and oligomers. Finally, the product was dissolved in chloroform and concentrated by rotary evaporation to obtain the final product.

Synthesis of Poly[3,3'''-bis(8-octylthiophenyl)quaterthiophene] (PQT-C8).

PQT-C8 (M_n = 18.9 kg/mol, PDI= 2.25) was synthesized by Stille copolymerization according to previously method.[3,4] 2TC8-2Br (466.8 mg), 2T-2Sn (419.0 mg), $Pd_2(dba)_3$ (31.3 mg) and P(*o*-tol)$_3$ (41.4 mg) were added into a microwave reaction flask, dissolved in 15 mL anhydrous chlorobenzene (CB) and fully stirred under the action of magneton. The reaction flask was continuously purged with nitrogen gas during the dissolution process to maintain an inert atmosphere. The flask was then placed in a microwave reactor, stirred for 1 min, reacted at 140 °C for 2 min, reacted at 160 °C for 2 min, and then reacted at 180 °C for 20 min. After the reaction, the solution was poured into a mixture containing 400 mL of methanol and 20 mL of hydrochloric acid, allowed to settle for 30 min, and then filtered to obtain the crude product. The polymer was purified using the Soxhlet extraction method, with methanol, acetone, n-hexane, and chloroform used in sequence to remove unreacted monomers and oligomers. Finally, the product was dissolved in chloroform and concentrated by rotary evaporation to obtain the final product.

Synthesis of Poly[3,3'''-bis(10-decylthiophenyl)quaterthiophene] (PQT-C10).

PQT-C10 (M_n = 20.6 kg/mol, PDI= 2.30) was synthesized by Stille copolymerization according to previously method.[3,4] 2TC10-2Br (513.7 mg), 2T-2Sn (418.9 mg), $Pd_2(dba)_3$ (31.0 mg) and P(*o*-tol)$_3$ (41.5 mg) were added into a microwave reaction flask, dissolved in 15 mL anhydrous chlorobenzene (CB) and fully stirred under the action of magneton. The reaction flask was continuously purged with nitrogen gas during the dissolution process to maintain an inert atmosphere. The flask was then placed in a microwave reactor, stirred for 1 min, reacted at 140 °C for 2 min, reacted at 160 °C for 2 min, and then reacted at 180 °C for 20 min. After the reaction, the solution was poured into a mixture containing 400 mL of methanol and 20 mL

of hydrochloric acid, allowed to settle for 30 min, and then filtered to obtain the crude product. The polymer was purified using the Soxhlet extraction method, with methanol, acetone, n-hexane, and chloroform used in sequence to remove unreacted monomers and oligomers. Finally, the product was dissolved in chloroform and concentrated by rotary evaporation to obtain the final product.

Density Functional Theory (DFT) Calculation.

Density functional theory (DFT) calculation was conducted using the Gaussian 16 program.[5] Molecular geometry optimization was performed with the ωB97xD/6-31G(d,p) basis set.[6]

Preparation of P3BT/PQT Blended Solutions and Films via the External Electric Field (EEF) Strategy.

P3BT was blended with PQT-C6/C8/C10 in a 1:1 mass ratio and dissolved in 1,2,4-trichlorobenzene (TCB) at a concentration of 5 mg/mL. During the EEF strategy, the polymer blended solution (100 μL) was dropped on a silicon substrate and confined between two copper electrode plates with a distance (d) of 5 mm. A direct current voltage (U = 0-7.5 kV) was applied between the two electrodes to form a parallel capacitor (Figure 1b). The electric field strength (E) is calculated from the following equation:

$$E = \frac{U}{d}$$

The EEF was applied continuously at room temperature for 6 h. The thickness of the formed films was in the range of 600-810 nm.

Calculation of Fractal Dimension (d_f) via Static Light Scattering (SLS).

The fractal dimension (d_f), a parameter which reflects the packing density of polymer chains in the solution, was determined via static light scattering (SLS). Higher d_f value means denser, more compact chain packing.[7] The scattered intensity $I_{(q)}$, where the scattering vector $q = 4\pi \sin(\theta/2)/\lambda$ (θ = 50°-150°, λ: incident wavelength), depends primarily on the particle form factor $P_{(q)}$ and the aggregate structure factor $S_{(q)}$. This relationship is expressed as:[7,8]

$$I_{(q)} = KP_{(q)}S_{(q)}$$

where K represents the optical constants of the instrument. In dilute solutions lacking interchain interactions, $S_{(q)} \approx 1$. For an aggregated system, the structure factor $S_{(q)}$ is directly related to the fractal dimension (d_f), which characterizes the compactness of the fractal aggregates.

Meanwhile, the scattering vector q is in the range of $R_{g,u} < q^{-1} < R_{g,agg}$, where $R_{g,u}$ and $R_{g,agg}$ denote the radius of gyration for the unimers and aggregates, respectively. The scattered light intensity $I_{(q)}$ is proportional to $S_{(q)}$ and q^{-d_f} as follows:[9]

$$I_{(q)} \propto S_{(q)} \propto q^{-d_f}$$

Consequently, d_f is derived from the slope of the linear fit to log $I_{(q)}$ versus log q.

Fabrication of Organic Field-Effect Transistors.

OFET devices with bottom-gate and top-contact structures were fabricated. Highly n-doped silicon wafers with a 300 nm SiO_2 layer were used as the gate electrode. Gold layer (~ 40 nm in thickness) was evaporated onto the polymer thin films as source/drain electrodes via a shadow mask with a channel length and width of 30 and 300 μm, respectively. The charge mobility (μ) was calculated from a transfer curve according to the following equation:[10]

$$I_{DS} = \frac{W}{2L}\mu C_g(V_G - V_T)^2$$

where I_{DS} is the drain current, C_g is the capacitance of SiO_2 dielectric (11.5 nF/cm^2), V_G is the gate voltage, and V_T is the threshold voltage.

Statistical Analysis.

All performance characterizations of organic field-effect transistors (OFETs) were based on at least ten independent devices. Data were expressed as mean ± standard deviation (SD) or shown with SD bars in the graphs.

Characterization.

^{1}H NMR spectra were obtained on a 600 MHz Bruker fully digital spectrometer using $CDCl_3$. Gel permeation chromatography (GPC) curves were performed on an Agilent 1260 Infinity II high-temperature system, using 1,2,4-trichlorobenzene (TCB) as the eluent at 160 °C and polystyrene standards for calibration. Two-dimensional grazing-incidence wide angle X-ray scattering (2D-GIWAXS) measurements were carried out at the BL02U2 beamlines (λ: 1.24 Å) at the Shanghai Synchrotron Radiation Facility (SSRF). X-ray diffraction (XRD) measurement was carried out on a Rigaku Smatlab 9KW diffractometer with a Cu Kα X-ray source (λ = 1.54 Å). A profilometry (AlphaStep D-600) was employed to measure the film thickness. Differential scanning calorimetry (DSC) was measured by the TA DSC250 at a heating rate of 10 °C/min under N_2 atmosphere. Cross-polarized optical microscopy (CPOM) pictures were captured by a Leica DM4M microscope. Atomic force microscopy (AFM) images

were acquired on an Oxford Cypher VRS 1250 in the tapping mode. Ultraviolet−visible (UV−vis) absorption spectra were measured using a PerkinElmer Lambda 750 UV-vis spectrophotometer. Light scattering (LS) data were obtained by a German-made dynamic/static wide-angle laser scattering instrument (ALV/CGS-3) with the operating wavelength of 632.8 nm. To avoid exceeding the measurement range of the instruments, the solutions were diluted to 1 mg/mL for UV-vis and LS characterization. OFET characteristics were measured using a Keithley 4200-SCS parameter analyzer in an argon-filled glovebox.

References.

1. A. Yokoyama, R. Miyakoshi, and T. Yokozawa, "Chain-Growth Polymerization for Poly(3-hexylthiophene) with a Defined Molecular Weight and a Low Polydispersity" *Macromolecules* **2004**, *37,* 1169-1171.

2. N. Doubina, A. Ho, A. K. Y. Jen, and C. K Luscombe, "Effect of Initiators on the Kumada Catalyst Transfer Polycondensation Reaction" *Macromolecules* **2009**, *42,* 7670-7677.

3. I. McCulloch, M. Heeney, C. Bailey, et al., "Liquid-crystalline semiconducting polymers with high charge-carrier mobility" *Nat. Mater.* **2006**, *5*, 328-333.

4. S. Y. Ku, M. A. Brady, N. D. Treat, et al., "A Modular Strategy for Fully Conjugated Donor-Acceptor Block Copolymers" *J. Am. Chem. Soc.* **2012**, *134*, 16040-16046.

5. Gaussian 16, Revision C.01, M. J. Frisch, G. W. Trucks, H. B. Schlegel, et al., *Gaussian, Inc.*, Wallingford CT, **2019**.

6. C. Adamo, and V. Barone, "Toward Reliable Density Functional Methods without Adjustable Parameters: The PBE0 Model" *J. Chem. Phys.* **1999**, *110*, 6158-6170.

7. S. Dai, K. C. Tam, and R. D. Jenkins, "Microstructure of Dilute Hydrophobically Modified Alkali Soluble Emulsion in Aqueous Salt Solution" *Macromolecules* **2000**, *33*, 404-411.

8. M. W. Ishaq, N. Hao, M. Zhu, and L. W. Li, "Light Scattering Study of Internal Motions of Ultralong Comb-like Chains in Dilute Solutions under Good Solvent Conditions" *Macromolecules* **2020**, *53*, 558-568.

9. T. Li, H. Zhang, B. Liu, et al., "Effect of Solvent on the Solution State of Conjugated Polymer P7DPF Including Single-Chain to Aggregated State Structure Formation, Dynamic Evolution, and Related Mechanisms" *Macromolecules* **2020**, *53*, 4264-4273.

10. S. Kim, H. Yoo, and J. Choi, "Effects of Charge Traps on Hysteresis in Organic Field-Effect Transistors and Their Charge Trap Cause Analysis through Causal Inference Techniques" *Sensors* **2023**, *23*, 2265.

Table S1. Summary of Molecular Weights and Polydispersity Index (PDI) of P3BT, PQT-C6, PQT-C8 and PQT-C10.

Polymers	M_n (kg/mol)	PDI
P3BT	3.7	1.43
PQT-C6	15.8	2.34
PQT-C8	18.9	2.25
PQT-C10	20.6	2.30

Table S2. Summary of 2D-GIWAXS Results for Four Homopolymers and Three P3BT/PQT Blended Films under Different Conditions.

Polymers	E [a] (kV/cm)	q_z [b] (nm^{-1})	d_{100} [c] (nm)	$FWHM_{100}$ [d] (nm^{-1})	d_{010} [e] (nm)
P3BT	0	6.48	0.97	0.23	0.42
	8	6.41/5.19	0.98/1.21	0.51/0.34	0.42/0.38
	15	5.15	1.22	0.31	0.38
PQT-C6	0	5.06	1.24	0.63	-
	8	5.19	1.21	0.60	-
	15	5.06	1.24	0.59	0.37
PQT-C8	0	4.24	1.48	0.59	-
	8	4.33	1.45	0.37	0.37
	15	4.39	1.43	0.28	0.37
PQT-C10	0	3.72	1.69	0.61	-
	8	3.83	1.64	0.54	0.37
	15	3.74	1.68	0.52	0.37
P3BT/PQT-C6	0	6.48/5.02	0.97/1.25	0.25/0.53	-
	8	4.91	1.28	0.35	-
	15	4.80	1.31	0.48	0.38
P3BT/PQT-C8	0	6.41/4.27	0.98/1.47	0.23/0.54	-
	8	4.91/4.39	1.28/1.43	0.27/0.53	0.38
	15	4.68	1.34	0.46	0.38
P3BT/PQT-C10	0	6.41/3.76	0.98/1.67	0.24/0.53	-
	8	4.95/4.03	1.27/1.56	0.33/0.37	0.38
	15	4.98/4.24	1.26/1.48	0.32/0.36	0.38

[a] The EEF strength. [b] Scattering vector of (100) peak along the out-of-plane (q_z) direction. [c] Lamellar distance in the (100) direction. [d] Full width at half-maximum (FWHM) of the (100) diffraction peak. [e] The π-π stacking distance in the (010) direction.

Table S3. Summary of Molecular Orientation in Three P3BT/PQT Blended Films under External Electric Field (EEF).

Polymers	*E* [a] (kV/cm)	edge-on (χ = 3.5-45°) content [b] %
P3BT/PQT-C6	0	75.5/73.6
	15	83.0
P3BT/PQT-C8	0	81.5/78.4
	15	81.4
P3BT/PQT-C10	0	83.4/91.2
	15	94.9/88.2

[a] The EEF strength. [b] Quantitative analysis of edge-on crystals within the χ range (χ: the angle between the crystal orientation and surface normal). The peak area (χ = 3.5–45°) region corresponds to the edge-on crystals.

Table S4. Summary of OFET Device Performances of Four Homopolymers and Three P3BT/PQT Blended Films Produced under Different Conditions.

Polymers	E [a] (kV/cm)	μ_{avg} [b] ($cm^2 V^{-1} s^{-1}$)	μ_{max} [c] ($cm^2 V^{-1} s^{-1}$)	V_{th} (V) [d]	I_{on}/I_{off} [e]
P3BT	0	$(6.25 \pm 0.58) \times 10^{-4}$	7.23×10^{-4}	-4.0 ~ 6.4	10^2
	15	$(1.63 \pm 0.23) \times 10^{-3}$	1.93×10^{-3}	-21.1 ~ -17.2	$10^2 \sim 10^4$
PQT-C6	0	$(1.03 \pm 0.19) \times 10^{-3}$	1.30×10^{-3}	-11.7 ~ -5.6	$10^2 \sim 10^4$
	15	$(2.15 \pm 0.31) \times 10^{-3}$	2.76×10^{-3}	-20.9 ~ -16.4	10^3
PQT-C8	0	$(1.44 \pm 0.09) \times 10^{-3}$	1.55×10^{-3}	-5.1 ~ 8.6	10^2
	15	$(2.60 \pm 0.11) \times 10^{-3}$	2.80×10^{-3}	-2.8 ~ 3.7	10^2
PQT-C10	0	$(1.60 \pm 0.11) \times 10^{-3}$	1.80×10^{-3}	-3.0 ~ 6.2	$10^1 \sim 10^2$
	15	$(3.19 \pm 0.15) \times 10^{-3}$	3.42×10^{-3}	-7.1 ~ -0.6	$10^2 \sim 10^3$
P3BT/PQT-C6	0	$(2.13 \pm 0.17) \times 10^{-3}$	2.51×10^{-3}	-20.9 ~ -15.5	$10^2 \sim 10^3$
	8	$(4.84 \pm 0.26) \times 10^{-3}$	5.13×10^{-3}	-27.2 ~ -23.2	$10^3 \sim 10^5$
	15	$(5.58 \pm 0.34) \times 10^{-3}$	6.07×10^{-3}	-28.3 ~ -22.6	$10^3 \sim 10^5$
P3BT/PQT-C8	0	$(1.73 \pm 0.18) \times 10^{-3}$	1.91×10^{-3}	-34.0 ~ -17.2	$10^3 \sim 10^5$
	8	$(2.43 \pm 0.18) \times 10^{-3}$	2.61×10^{-3}	-26.4 ~ -14.9	$10^3 \sim 10^5$
	15	$(4.42 \pm 0.14) \times 10^{-3}$	4.55×10^{-3}	-22.9 ~ -20.7	$10^3 \sim 10^5$
P3BT/PQT-C10	0	$(1.47 \pm 0.24) \times 10^{-3}$	1.93×10^{-3}	-20.3 ~ -11.0	$10^2 \sim 10^4$
	8	$(2.17 \pm 0.20) \times 10^{-3}$	2.43×10^{-3}	-21.1 ~ -17.3	$10^2 \sim 10^3$
	15	$(2.40 \pm 0.13) \times 10^{-3}$	2.64×10^{-3}	-25.5 ~ -19.6	$10^2 \sim 10^3$

[a] The EEF strength. [b] Average charge mobility acquired from at least 10 transistors. [c] Maximum charge mobility. [d] Threshold voltage. [e] Current on/off ratio.

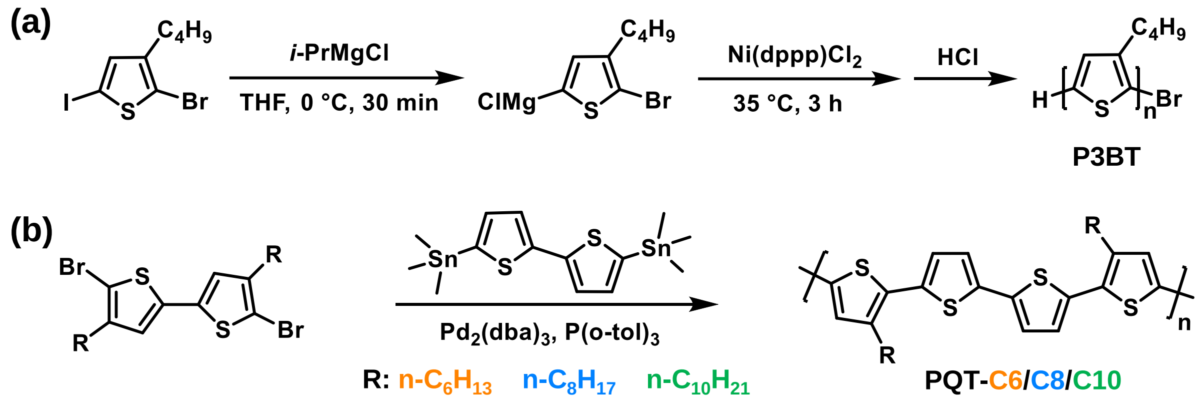

Figure S1. Synthesis routes of (a) P3BT and (b) PQTs (i.e., PQT-C6, PQT-C8, and PQT-C10) with different alkyl side chains.

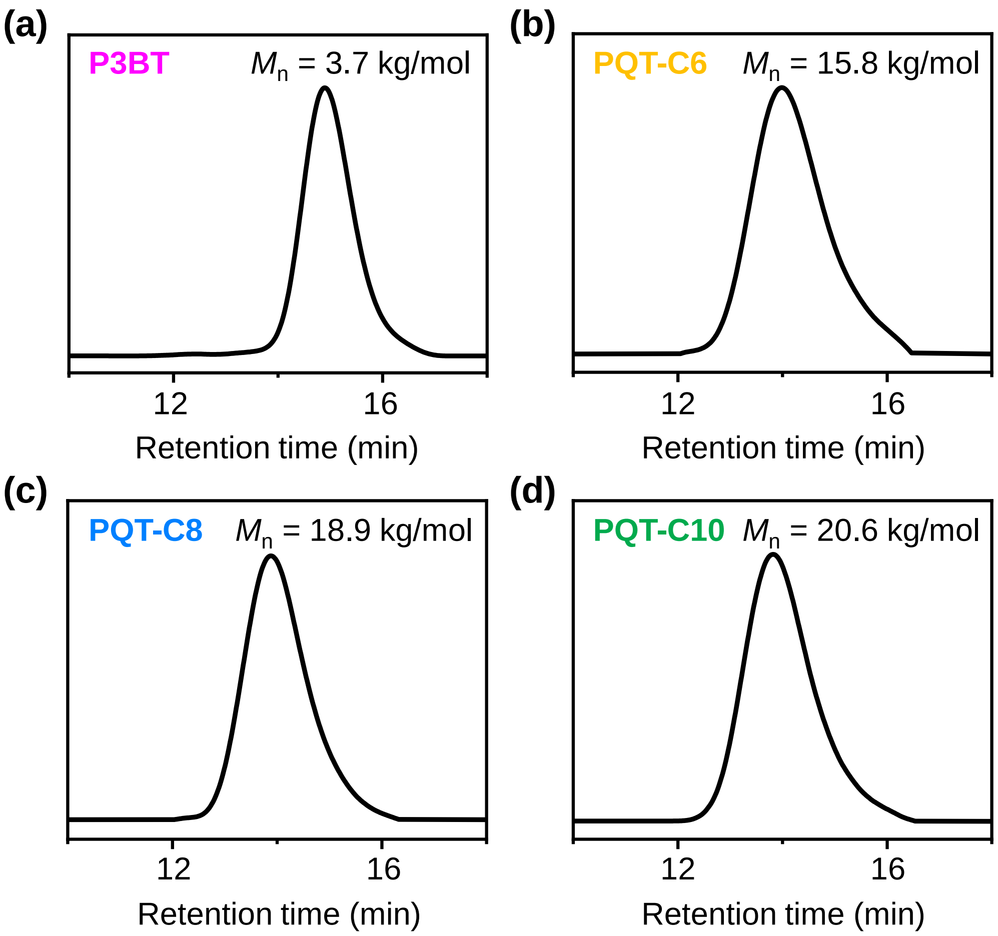

Figure S2. GPC profiles of (a) P3BT (M_n = 3.7 kg/mol), (b) PQT-C6 (M_n = 15.8 kg/mol), (c) PQT-C8 (M_n = 18.9 kg/mol) and (d) PQT-C10 (M_n = 20.6 kg/mol).

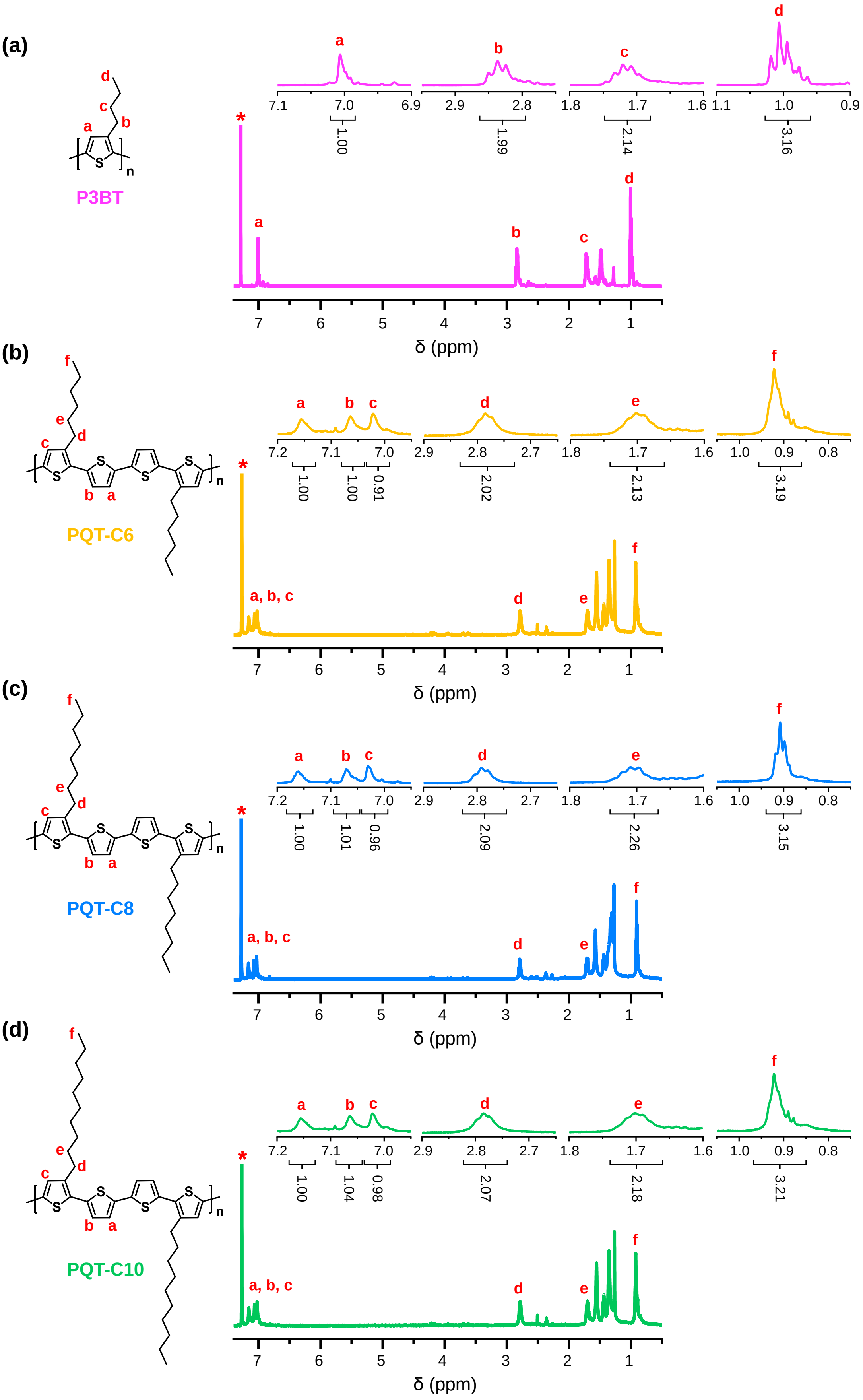

Figure S3. ^{1}H NMR spectra of (a) P3BT, (b) PQT-C6 (c) PQT-C8 and (d) PQT-C10 in $CDCl_3$ (*).

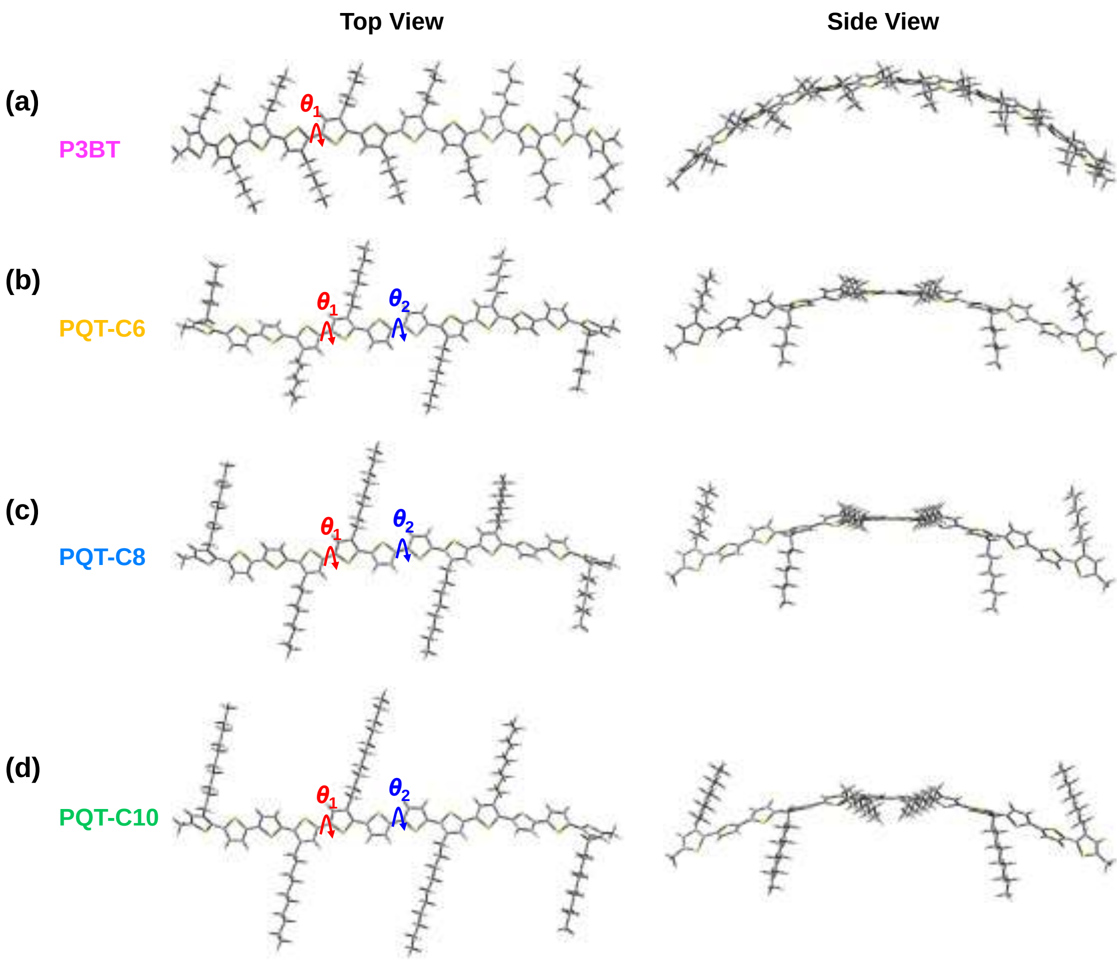

Figure S4. DFT-simulated geometries of the dodecamers of (a) P3BT and the trimers of (b) PQT-C6, (c) PQT-C8, (d) PQT-C10 in both top view (left) and side view (right).

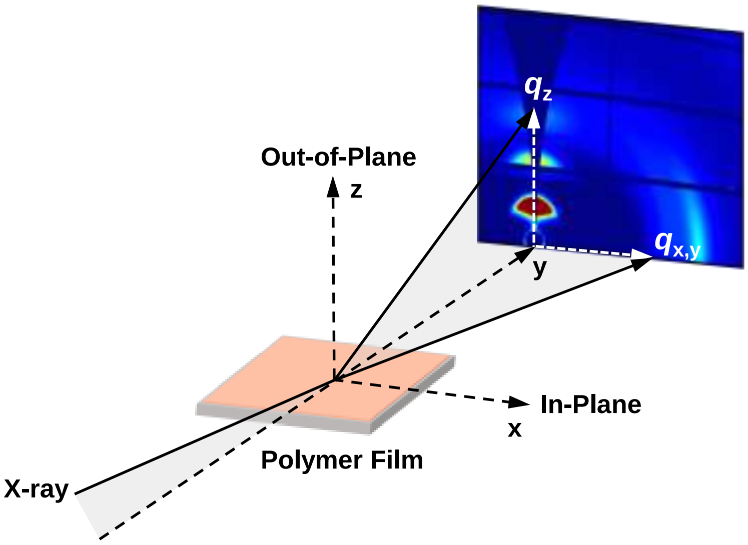

Figure S5. Schematic representation of two-dimensional grazing incidence wide angle X-ray scattering (2D-GIWAXS) experiment on the conjugated polymer film.

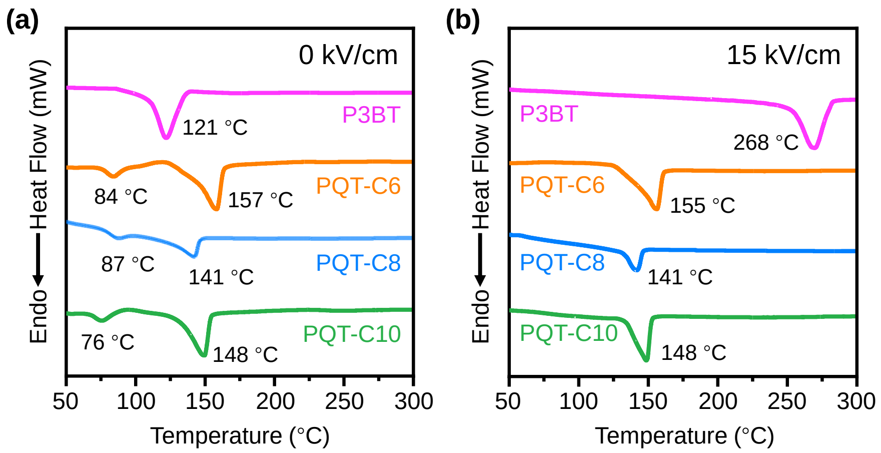

Figure S6. (a, b) DSC curves of four homopolymers (P3BT, PQT-C6, PQT-C8, and PQT-C10) films produced at (a) E = 0 kV/cm and (b) E = 15 kV/cm, respectively.

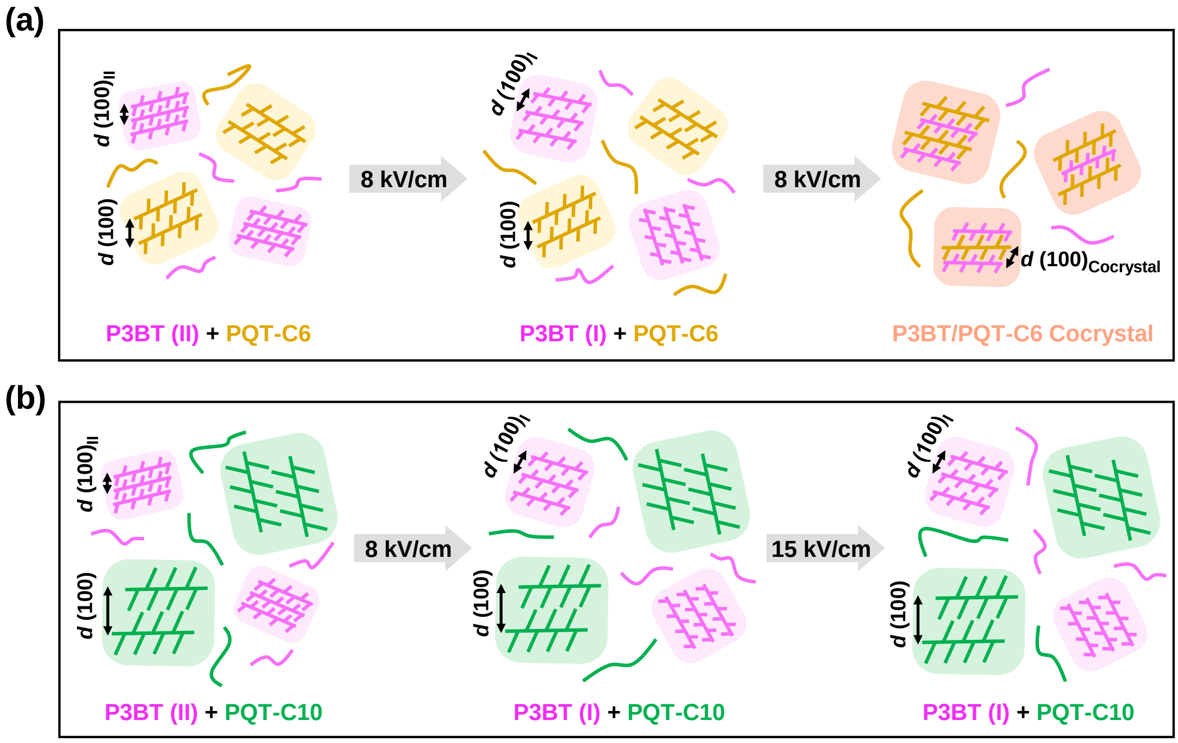

Figure S7. (a) Schematic of the crystalline structure evolution in the P3BT/PQT-C6 blend during the EEF process. At 0 kV/cm, P3BT in polymorph II (pink domain) and PQT-C6 (yellow domain) phase-separate with each other and form respective crystal domains (left panel). P3BT transforms into polymorph I at 8 kV/cm (middle panel), and then form cocrystals (orange domain) at this EEF strength (8 kV/cm), in which their alkyl side chains interdigitate with each other and produce a single d_{100} (right panel). (b) Schematic of the crystalline structure evolution in the P3BT/PQT-C10 blend during the EEF process. At 0 kV/cm, P3BT in polymorph II (pink domain) and PQT-C10 (green domain) phase-separate with each other and form respective crystal domains (left panel). P3BT transforms into polymorph I and phase-separate with PQT-C10 at 8 kV/cm (middle panel). They remain phase separation when E is further increased to 15 kV/cm (right panel).

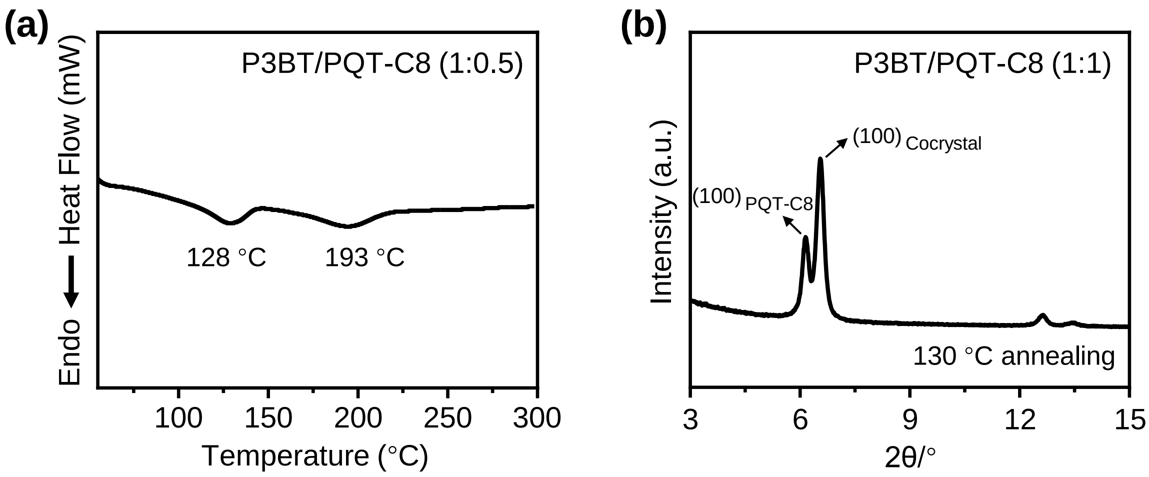

Figure S8. (a) DSC curves of P3BT/PQT-C8 (1:0.5) blend under the EEF strength of 15 kV/cm. (b) XRD profiles of P3BT/PQT-C8 (1:1) blend after thermal annealing at 130 °C.

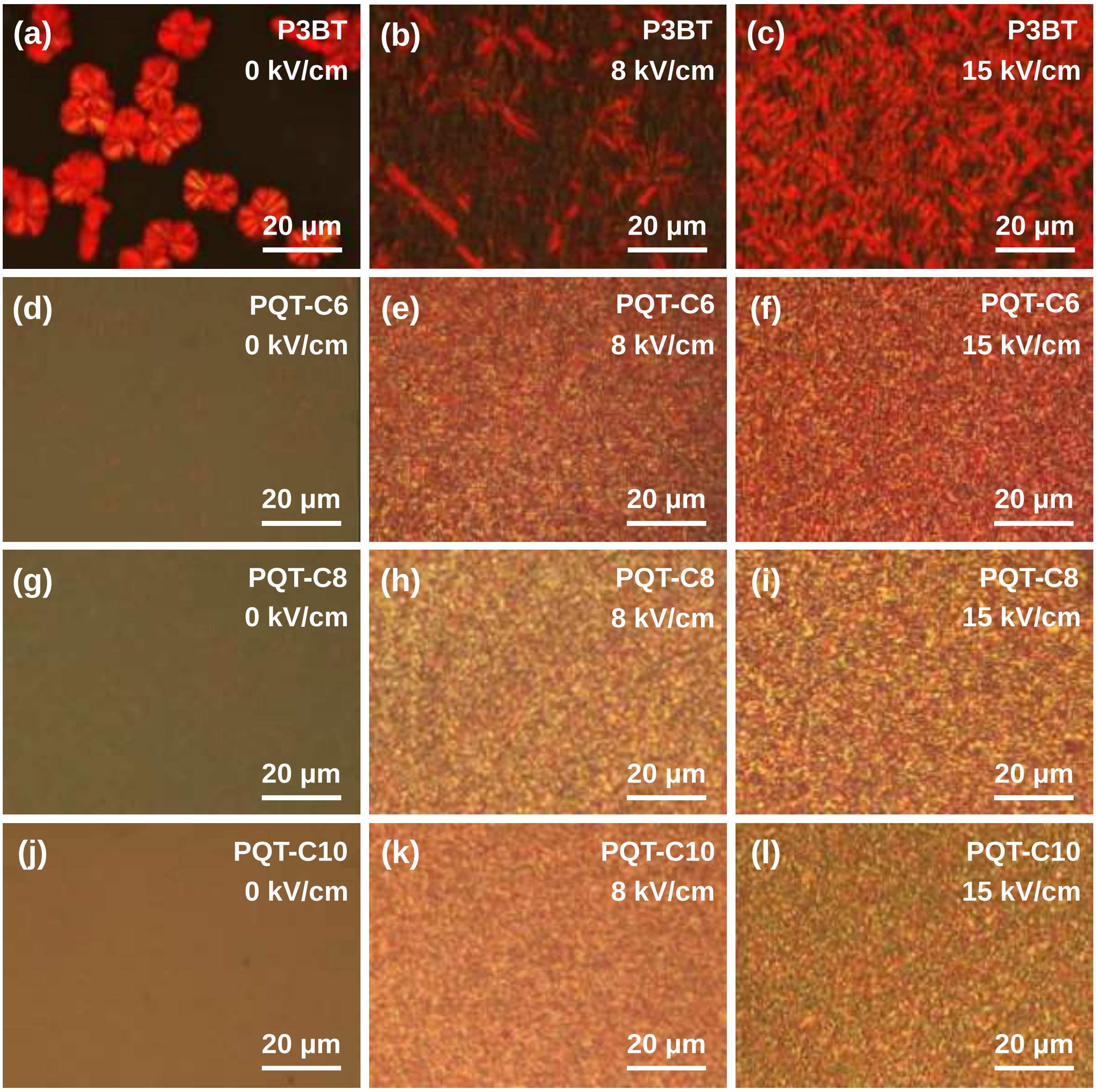

Figure S9. (a-l) CPOM images of (a-c) P3BT, (d-f) PQT-C6, (g-i) PQT-C8, (j-l) PQT-C10 films produced under different EEF strengths.

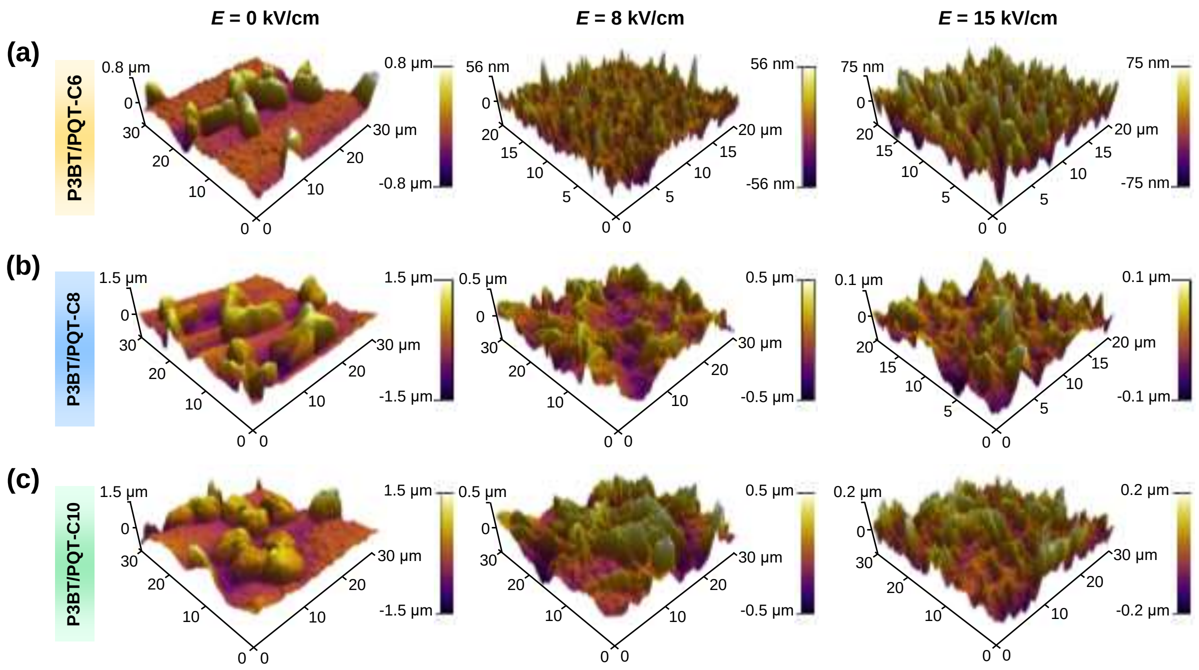

Figure S10. The AFM topography images of three P3BT/PQT blended films: (a) P3BT/PQT-C6, (b) P3BT/PQT-C8, and (c) P3BT/PQT-C10 at different EEF strengths (0, 8, and 15 kV/cm).

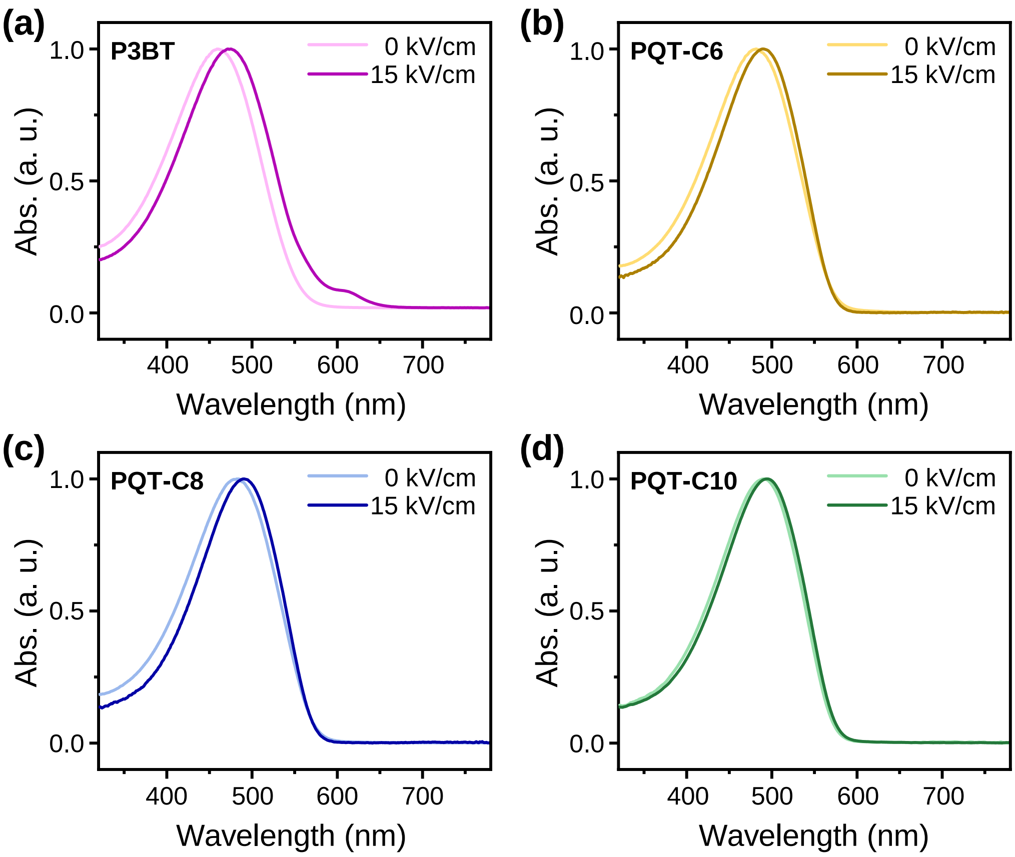

Figure S11. (a-d) UV-vis absorption spectra of (a) P3BT, (b) PQT-C6, (c) PQT-C8, (d) PQT-C10 solutions under different EEF strengths.

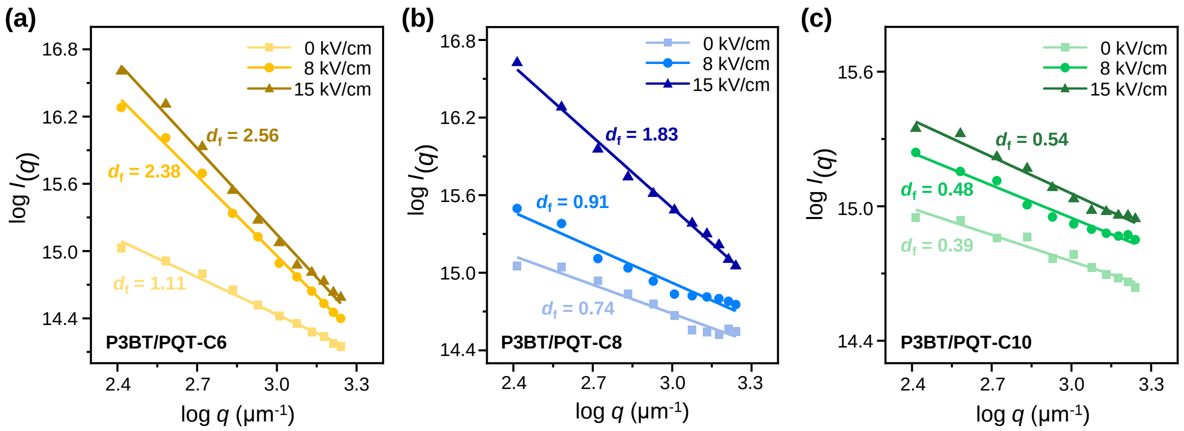

Figure S12. Plots of log $I_{(q)}$ versus log q for (a) P3BT/PQT-C6, (b) P3BT/PQT-C8 and (c) P3BT/PQT-C10 blends in the solution at different EEF strengths. The fractal dimension (d_f) of the solution aggregates is obtained from the slop of the log $I_{(q)}$ versus log q after linear fitting.

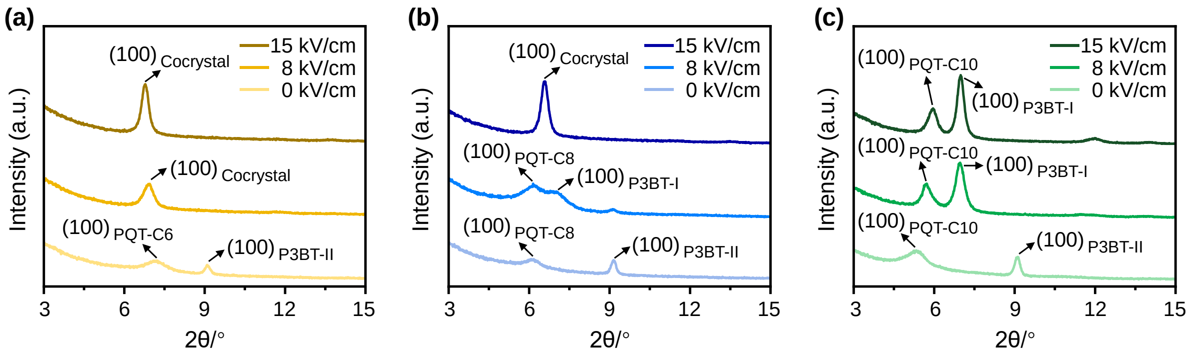

Figure S13. XRD profiles of three P3BT/PQT blended films (1 mg/mL) produced under different EEF strengths (0, 8 and 15 kV/cm): (a) P3BT/PQT-C6, (b) P3BT/PQT-C8 and (c) P3BT/PQT-C10.

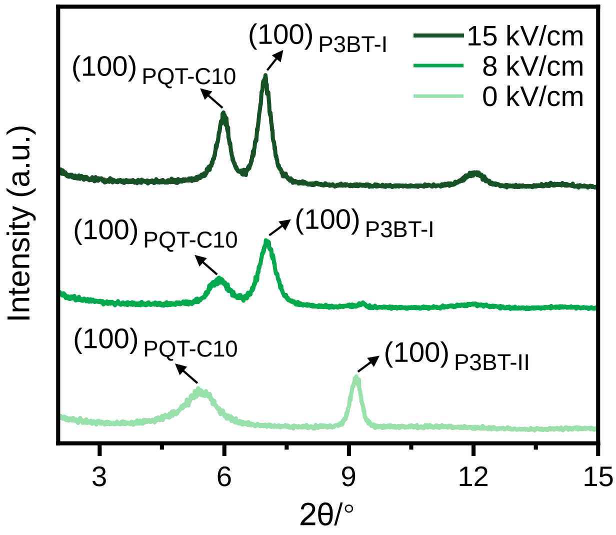

Figure S14. XRD profiles of P3BT/PQT-C10 blended films produced under different EEF strengths (0, 8 and 15 kV/cm), in which the PQT-C10 had a lower M_n of 11.1 kg/mol.

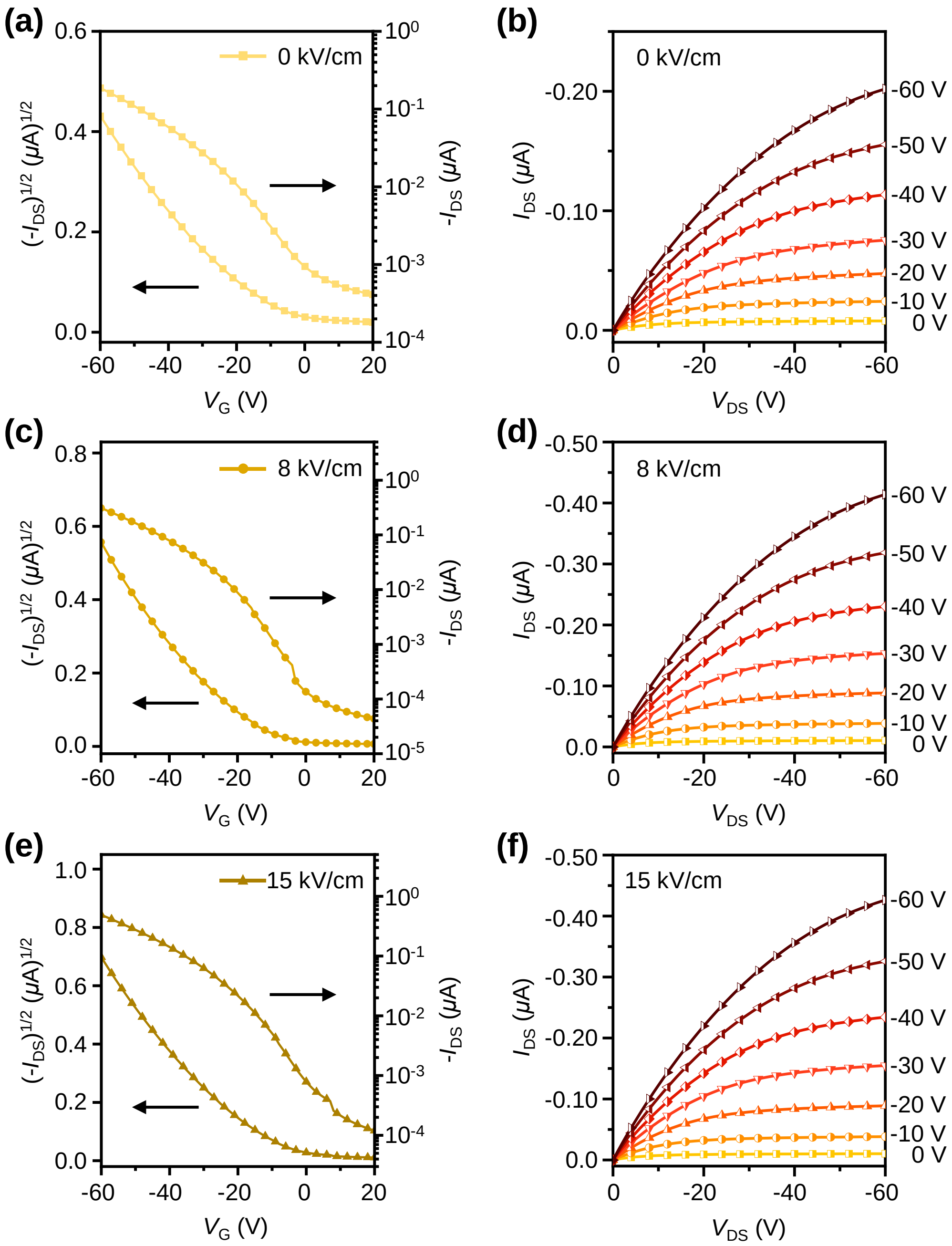

Figure S15. (a, c, e) Transfer and output (b, d, f) curves of P3BT/PQT-C6 blended films formed at various EEF strengths.

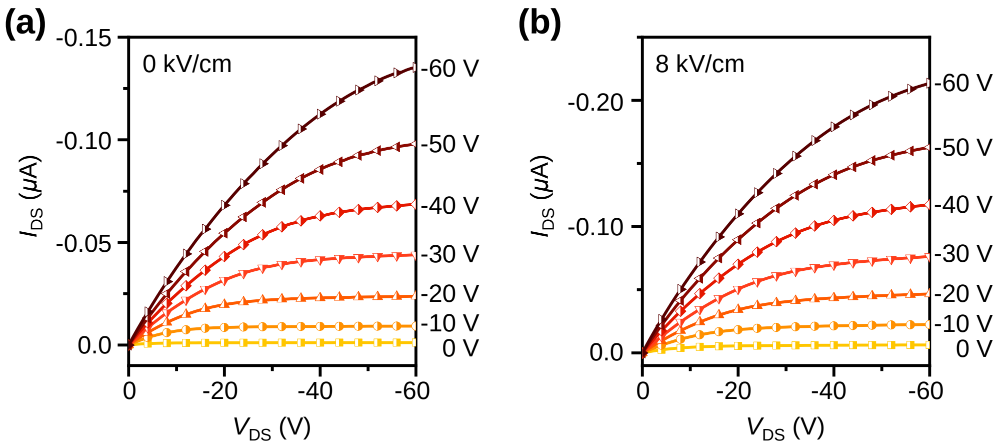

Figure S16. (a, b) Output curves of P3BT/PQT-C8 blended films formed at various EEF strengths.

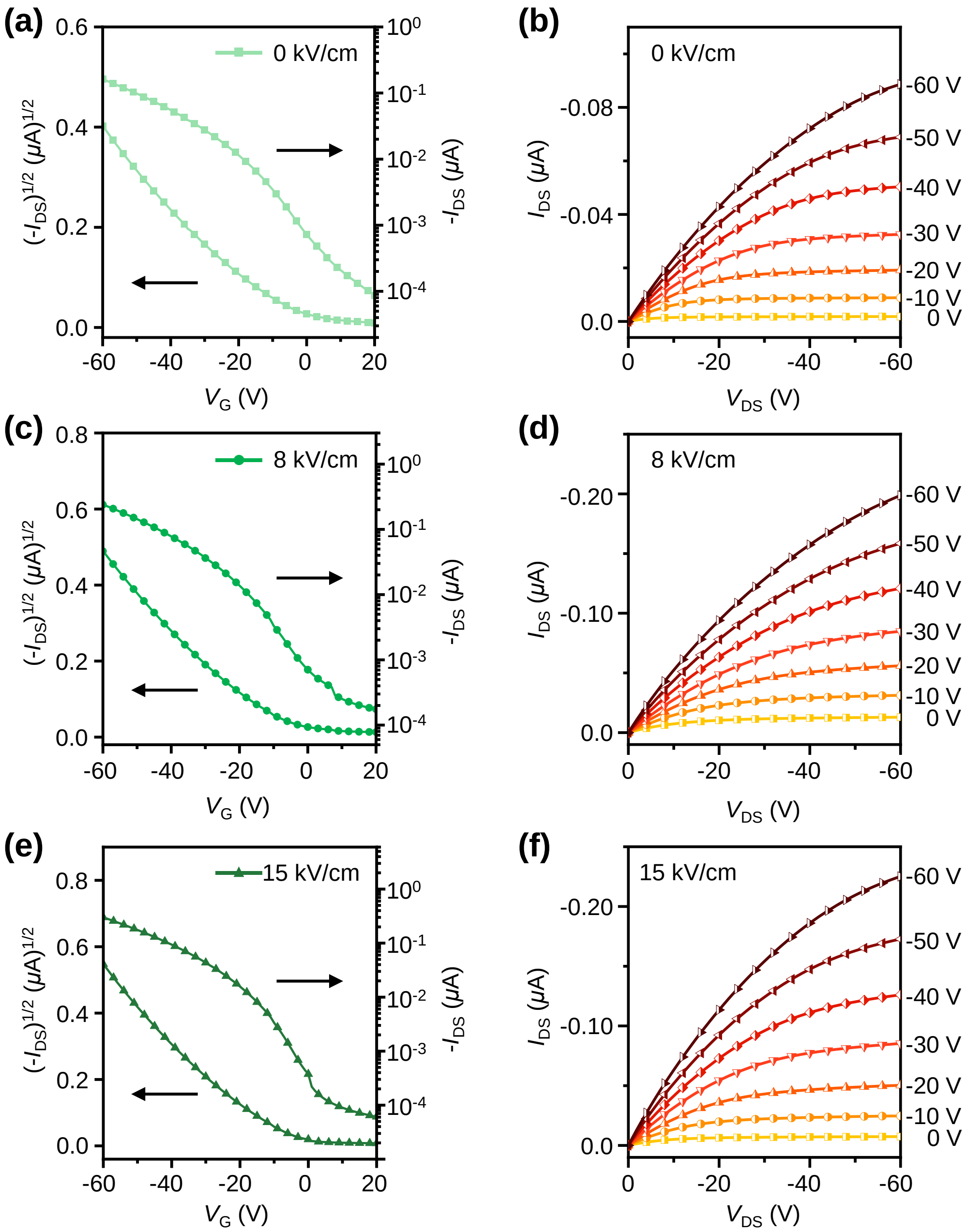

Figure S17. (a, c, e) Transfer and output (b, d, f) curves of P3BT/PQT-C10 blended films formed at various EEF strengths.

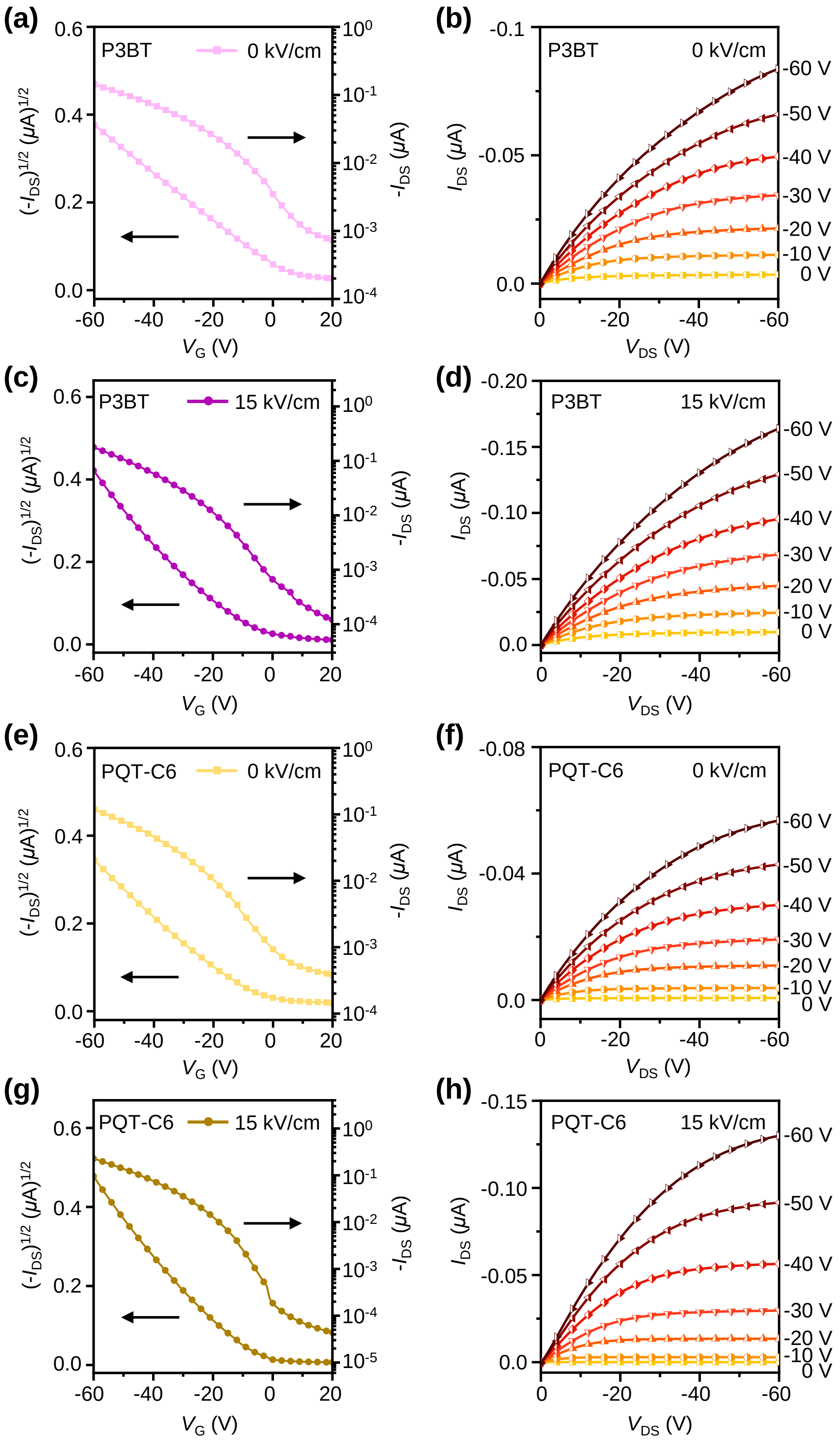
(a)
P3BT
0 kV/cm
$(-I_{DS})^{1/2}$ (μA)$^{1/2}$
$-I_{DS}$ (μA)
V_G (V)
(b)
P3BT
0 kV/cm
I_{DS} (μA)
V_{DS} (V)
-60 V
-50 V
-40 V
-30 V
-20 V
-10 V
0 V
(c)
P3BT
15 kV/cm
(d)
P3BT
15 kV/cm
(e)
PQT-C6
0 kV/cm
(f)
PQT-C6
0 kV/cm
(g)
PQT-C6
15 kV/cm
(h)
PQT-C6
15 kV/cm

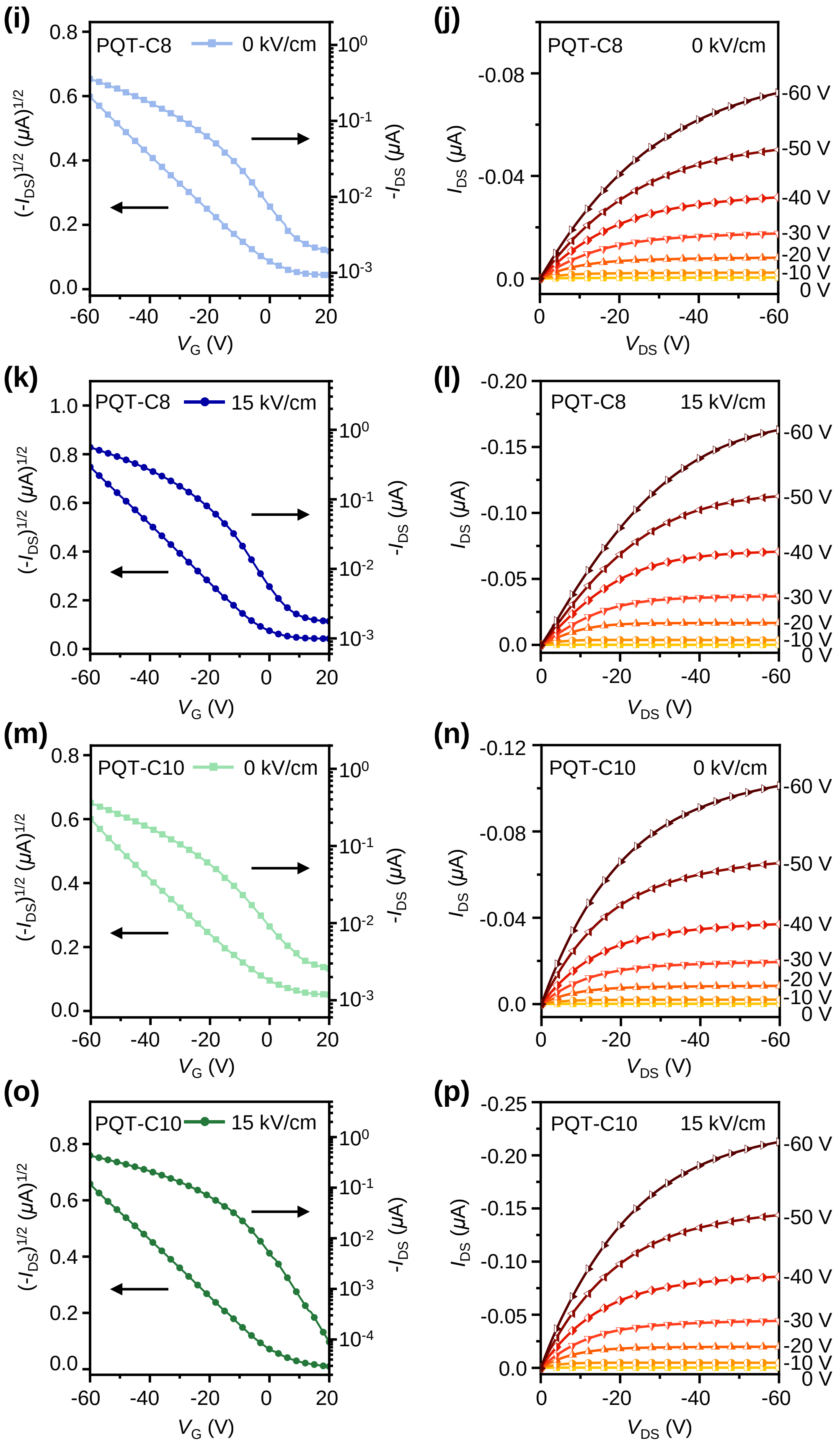

Figure S18. (a, c, e, g, i, k, m, o) Transfer and (b, d, f, h, j, l, n, p) output curves of (a-d) P3BT, (e-h) PQT-C6, (i-l) PQT-C8, and (m-p) PQT-C10 films formed at various EEF strengths.
